# Shallow-Water Northern Hemisphere *Jaera* (Crustacea, Isopoda, Janiridae) Found on Whale Bones in the Southern Ocean Deep Sea: Ecology and Description of *Jaera tyleri* sp. nov

**DOI:** 10.1371/journal.pone.0093018

**Published:** 2014-03-24

**Authors:** Katrin Linse, Jennifer A. Jackson, Marina V. Malyutina, Angelika Brandt

**Affiliations:** 1 British Antarctic Survey, Cambridge, United Kingdom; 2 A.V. Zhirmunsky Institute of Marine Biology, Far Eastern Branch, Russian Academy of Sciences, Vladivostok, Russia; 3 Biocentre Grindel and Zoological Museum, University of Hamburg, Hamburg, Germany; Heriot-Watt University, United Kingdom

## Abstract

The skeleton of a natural whale fall discovered in the Southern Ocean at 1,445 m was densely covered by one small, janirid isopod. *Jaera tyleri* sp. nov. is the first of its genus found in the southern hemisphere and in the deep sea and is described herein. Morphological and molecular investigations revealed the systematic position of this species new to science. Phylogenetic analysis of the 18S locus confirms that this species falls in a strongly supported monophyletic clade of *Jaera* species. The whale bone habitat of *J. tyleri* sp. nov. is quite different from those of other species of the genus *Jaera*. The analysis of bathymetric and distribution patterns of the Janiridae in general and *Jaera* specifically confirm the unusualness of the habitat for this isopod species. The abundance of *J. tyleri* sp. nov. on the whale bones and its absence from other nearby habitats suggests it to be a whale-fall specialist. The analysis of the size-frequency distributions of *J. tyleri* sp. nov. suggests multimodal population structure with continuous breeding activity throughout the year. The fecundity of the species is low but in line with reduced fecundity observed in polar and small-sized isopods.

## Introduction

Until the discovery of whale falls as habitats of rich chemoautotroph communities in 1987 [1], studies of marine fauna discovered on dredged up whale bones have been more anecdotal and included taxonomic descriptions of the species [2], [3] and the aspect of whale bones as organic food sources [4]. Since then, both natural and experimentally-implanted whale carcasses have been subject of research on the degradation of the carcass, the food enrichment of the deep-sea environment and its effects on the surrounding benthic biodiversity [1], [5]–[8] Smith and Baco [5] defined three succession stages in the decay of whale falls, which attract different types of scavengers and food specialists. During the first stage, the *mobile-scavenger stage*, soft tissues are removed by mobile vertebrate and invertebrate scavengers like sleeper sharks, hagfish, cirolanid isopods, lysianassid amphipods or lithodid crabs [5], [9]. The second, *enrichment-opportunistic stage* is characterised by dense assemblages of heterotrophic, often invertebrate fauna, feeding on the remaining soft tissues and bones [5], [10]. The third stage, the *sulphophilic* stage is distinguished by diverse, trophically complex assemblages living on the skeleton, including whale-bone feeders, bacterial grazers, species utilising chemoautotrophic endosymbionts, deposit feeders, facultative suspension feeders and predators [5]. The whale skeletons emit sulphides from the anaerobic breakdown of bone lipids, which form the basis of a chemoautotrophic environment that is host to rich and abundant assemblage of specialists, like the bone eating polychaetes (*Osedax* spp.) and gastropods (*Pyropelta* spp.), the bivalve *Idas washingtonia* (Bernard 1978) or the isopod *Ilyarachna profunda* Schultz 1966 [5], [11].

The presence of isopod species at whale falls has been rarely reported, as research often focussed on the fish, decapod, gastropod, bivalve and polychaete species [5], [6], [9], [12], [13]. The giant cirolanid isopod *Bathynomus giganteus* Milne Edwards 1879 has been observed and filmed at relatively new whale falls, scavenging on the soft tissues [14]. The munnopsid Ilyarachna profunda has been recorded at whale skeletons in population sizes of 500–1800 [5] and appears to be the most numerous isopod species on whale falls. Smith and Baco [5] also mention the presence of family Janiridae at whale falls from southern California but do not specify the generic or species affiliation.

The marine isopod family Janiridae is globally distributed with records spanning from the Arctic to the Antarctic and from the Atlantic, Indian and Pacific oceans [15]. At present 174 species of the 23 genera are assigned to the Janiridae, although previous studies have shown that this family is not monophyletic and requires taxonomic revision [16], [17] These species occur over a wide range of habitats, including the intertidal, estuarine areas, salt springs, anchialine caves and on whale carcasses [5], [15] and from the intertidal to the hadal deep sea (e.g. [18], [19], [20]). Several of the intertidal and shallow water janirid species are herbivores and grazers, eating seaweed, dead wood and bacterial films; feeding strategies of the deep-water species are unknown [21]–[27]. Species of the Janiridae are known to be highly adjustable to their environments, including wide tolerances to salinity, temperature and oxygen stresses [28], [29]. The genus *Jaera* with its main distribution in the northern hemisphere has been subject to several ecological and reproductive studies [30]–[35].

Here we describe the first deep-water, bathyal species of the genus *Jaera*, found on a natural Antarctic whale fall, present information on its ecology and review the depth and biogeographic distribution patterns of *Jaera* and the Janiridae in general.

## Methods

### Ethics statement

All necessary permits were obtained for the described field studies. Studies in the East Scotia Sea were undertaken under the permit S3-3/2009 issued by the Foreign and Commonwealth Office, London to section 3 of the Antarctic Act 1994.

### Study site

**Figure 1:**
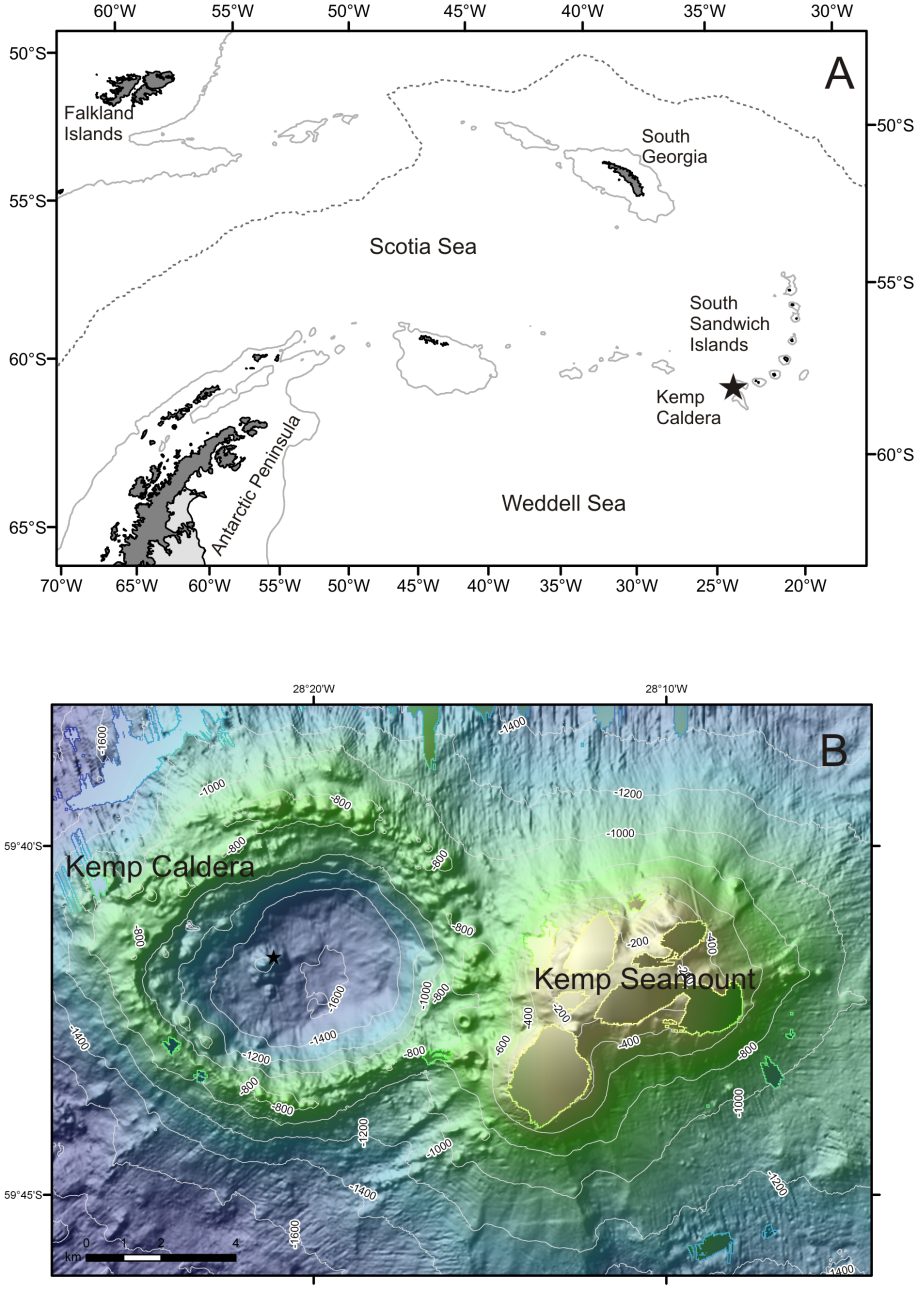
Map of Southern Ocean. A) Location of Kemp Caldera in Southern Ocean; B) Type locality in Kemp Caldera

**Figure 2:**
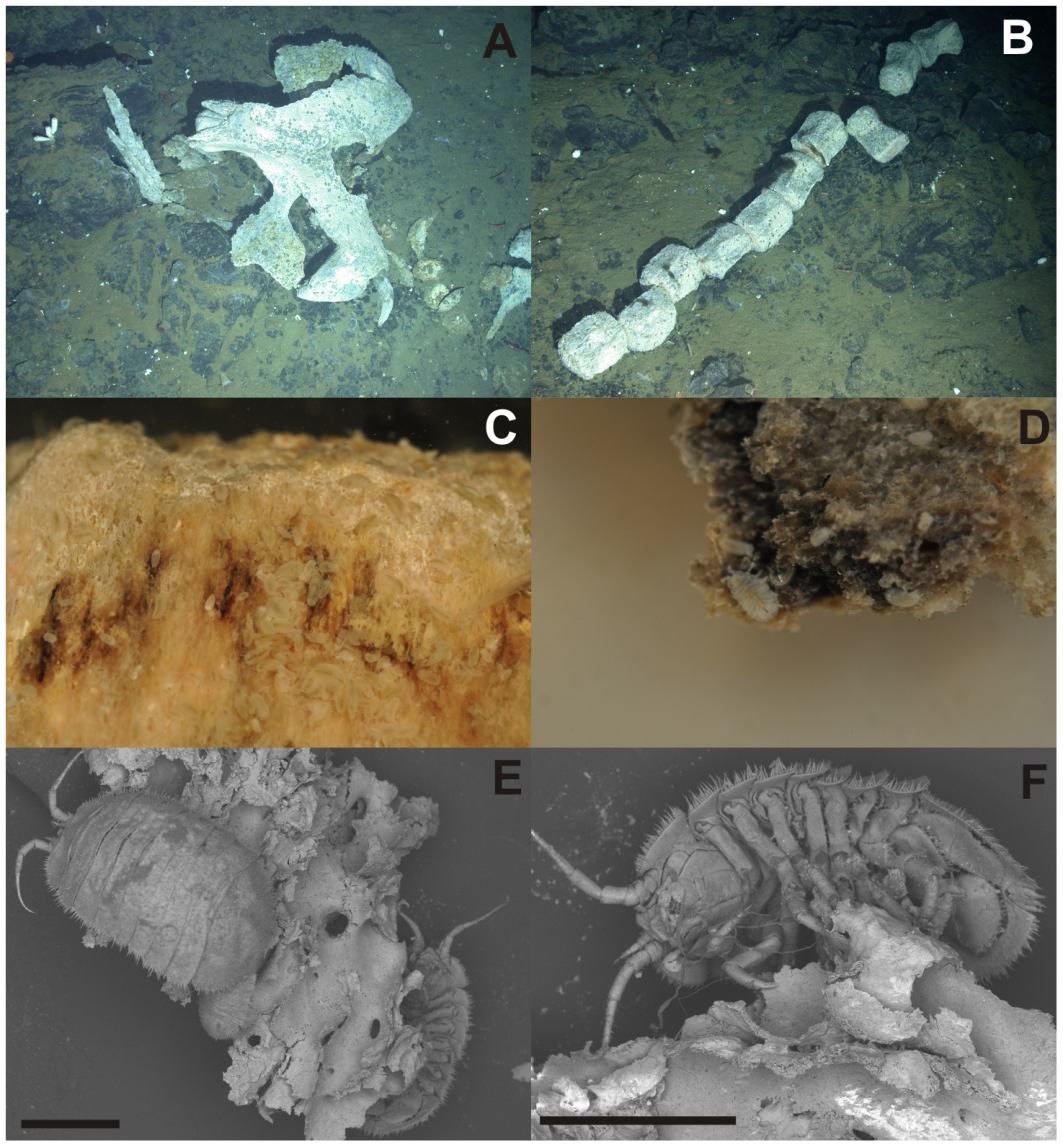
Kemp Caldera whale fall. A) Skull and jaws in situ; B) vertebrae in situ; C) whale bone with *Jaera tyleri* sp. nov.; D) whale bone with *Jaera tyleri* sp. nov.; E) *Jaera tyleri* sp. nov. on whale bone; F) *Jaera tyleri* sp. nov. on whale bone

During the expedition JC 42 of the RRS James Cook a baleen whale skeleton was discovered during dive 148 in a caldera next to the Kemp Seamount (59°41.6 S, 28°21.1 W (DDM)) in 1,444 to 1,447 m water depth (Fig. 1) in the vicinity of the South Sandwich Islands [8], [36]. The whale fall was examined using the imaging systems of the remote operating vehicle (ROV) *Isis*, of the National Oceanography Centre Southampton, a Scorpio digital still camera with flash unit was used as well as two high definition video cameras (1080i) (Fig. 2A, B).

### Morphological analysis

Specimens were collected by picking up whale bones with the seven-function Kraft Predator arms of ROV *Isis*, which were stored in a biobox. At arrival on deck the whale bones were transferred to the 4°C lab and photographed (Fig. 2C, D) before epifaunal sampling began. Isopods were either picked by forceps or decanted from bottom of the sorting trays, fixed in pre-cooled 96% ethanol or 4% buffered formaldehyde and the latter after 3 months transferred to 70% ethanol. The material was sorted in the laboratory, measured and identified using a Leica MZ 12 stereomicroscope equipped with a *camera lucida*, and drawn using an Olympus SZX7 compound microscope, also equipped with a *camera lucida*. For scanning electron microscopy (SEM), whole animals were vibrated in single vials for 30 sec in soapy water, rinsed first with distilled water, than 96% ethanol and air dried. The specimens on the whale bone piece were air dried only. The specimens were mounted on aluminium stubs and viewed uncoated in a Hitachi TM3000 (Fig. 2E, F).

The terminology and measurements mostly follow Wilson [37]. Total body length was measured medially from the tip of the rostrum to the posterior tip of the pleotelson. The dorsal view was used for measuring the width, while the length of body segments was measured in lateral view. For the description of the body and pereopods the holotype was used, and for mouthparts and pleopods a male paratype was dissected and for some details that the paratype lacked or were different from the male, a female paratype was used.

### Molecular analysis

Genomic DNA was isolated from isopod pereopods. DNA was extracted with the DNeasy Tissue Extraction Kit (Qiagen, Crawley, West Sussex, United Kingdom) as directed by the manufacturer. 18S reactions were performed in 10 μl volumes, containing 0.5 μl of each primer (forward and reverse) at a concentration of 10 nmol, 5 μl of Qiagen 10× PCR buffer, 1.5 μl of MgCl_2_ (25 mM), 1 μl dNTPs (2 nmol, Bioline), 0.25 μl of Taq (5 U/μl) and 1 μl of DNA template (∼ 30 ng). 18S rDNA was amplified using SSUA NSF4 (5′-CTGGTTGATYCTGCCAGT-3′) [38] and SSUA NSR581 (5′ATTACCGCGGCTGCTGGC-3′) [39] under the following conditions: Initial denaturation at 96°C for 0.5 minutes, followed by 40 cycles of 94°C for 0.5 min, 55°C for 0.5 min, 72°C for 1 min, and a final extension of 5 min at 72°C.

#### DNA sequencing was performed at LGC Berlin Germany

All sequences were edited and aligned in CodonCode Aligner Version 3.5.6 (CodonCode Corporation 2006). Sequence quality was evaluated using “Phred” quality scores, excluding sequences with values <300 [40], [41]. Electropherograms were also manually examined for sequencing errors and, where possible, variable positions were confirmed by reference to the corresponding reverse sequences. Close relatives of the sequences were determined using the ‘Blast’ searching tool in GenBank and revealed closest matches with asellote isopod species reported in Raupach et al. [16]. The *18*S fragments were aligned with additional *Jaera* species, *Jaera albifrons* (AF279609) and *J. nordmannii* (AF279610), as well as the 18S alignment obtained from Raupach et al. [16]. The whole fragment of 18S was analysed (2,302 bp) as well as a dataset truncated to the length of the *J. tyleri* fragment (680 bp). Outgroups from the Stenetriidae family were used, following Raupach et al. [16]. The 18S alignment was constructed using RNA Salsa v0.8.1 [42] after generating a ‘guide’ secondary structure using the ‘mfold’ web server (http://mfold.rna.albany.edu/?q=mfold). This analysis uses a thermodynamic folding algorithm to align DNA with respect to inferred stem and loop regions. Matching, mismatching and gap opening penalties were applied in this analysis with a default stringency of 0.6.

### Molecular Phylogenetic Analysis

The 18S alignment was subjected to partitioned maximum likelihood (ML) analysis with RaXML v7.4.2 [43], using a mixed model to account for different evolutionary processes occurring in the stem and loop regions. A standard general time reversible DNA substitution model with a gamma correction for rate heterogeneity was applied to the loop regions. Stems were analysed using multi-state secondary structure models which allow for 6–16 movement probabilities between different paired states (RaXML models 6A-D, 7A-D and 16A-D, [44]). ML analyses were conducted ten times for each model choice. Models were then compared using Akaike Information Criterion (AIC) scores, calculated from the log-likelihood values and numbers of free parameters (P) for each model (AIC = −2LnL+2P). The model with the smallest AIC score was then chosen and a maximum likelihood bootstrap was conducted with 1000 replicates.

### Biogeographic and depth analysis

Data on bathymetric and georeferenced distributions of the Janiridae were obtained from original literature references. The isopod world list [15] http://www.nmnh.si.edu/iz/isopod/, www.gbif.org) was used but with *Jaera* taxonomy edited following Harvey and Naylor [45], Tobias et al. [46] and Borza [47]. It is clear that because data are pooled from a variety of sources, not all taxa will necessarily occur together at one depth, however, they have at least been reported at a single location at that depth interval. For the distribution analysis of *Jaera* only species confirmed records were used.

For the analysis of bathymetric distribution patterns, water depth was divided into 100 m wide depth intervals. For the account of species numbers per depth the number of species found in the depth interval was taken. For the assessment of the depth range at genus level, the presence at each depth interval of species of the selected genus was taken. When breaks occurred in the depth distributions of two or more species within a family, a break is recorded in the results. The term bathyal was used for stations ranging from ∼ 1,000 m down to 3,000 m, abyssal from ∼ 3,500 – 6,000 m where the hadal starts [48].

### Ecological analysis

A representative subset of paratypes was measured, growth stages and sex identified using Leica MZ 12 and Zeiss Stemi SV6 stereomicroscopes equipped with eyepiece micrometers. Total body length was measured to the nearest 0.1 mm. Growths stages were separated following morphological characteristics into Manca I (6 pereomeres, 6 legs, tiny 7th pereonite), Manca II (small 7^th^ pereonite, short 7^th^ leg), immature male (short, not fully developed 1^st^ pleopod), male (fully developed 1^st^ pleopod), immature female (1^st^ pleopod absent, 2^nd^ pleopod formed the operculum, no oostegites present), female (oostegites present) and ovigerous female (eggs in brood pouch). If present, numbers of eggs in brood pouch were counted.

### Abbreviations

An1 – antennula, An2 – antenna 2, lMd – left mandible, rMd – right mandible, Mx1 – maxilla 1, Mx2 – maxilla 2, Mxp – maxilliped, Hy – hypopharynx, P1–7 – pereopods 1–7, Plp 1–5 – pleopods 1–5, Ur – uropod.

### Nomenclatural Acts

The electronic edition of this article conforms to the requirements of the amended International Code of Zoological Nomenclature [49], and hence the new name contained herein is available under that Code from the electronic edition of this article. This published work and the nomenclatural act it contains have been registered in ZooBank, the online registration system for the ICZN. The ZooBank LSIDs (Life Science Identifiers) can be resolved and the associated information viewed through any standard web browser by appending the LSID to the prefix “http://zoobank.org/”. The LSID for this publication is: urn:lsid:zoobank.org:pub: 112A7E66-FB6E-4821-A2AA-52E28156CFC0 and for Jaera tyleri sp. nov. is: urn:lsid:zoobank.org:act urn:lsid:zoobank.org:act: 2B77D993-22F1-4EB0-906A-7D210E3E0437 The electronic edition of this work was published in a journal with an ISSN, and has been archived and is available from NERC's Open Research Archive NORA.

## Results

### Distribution patterns in *Janiridae*

**Figure 3:**
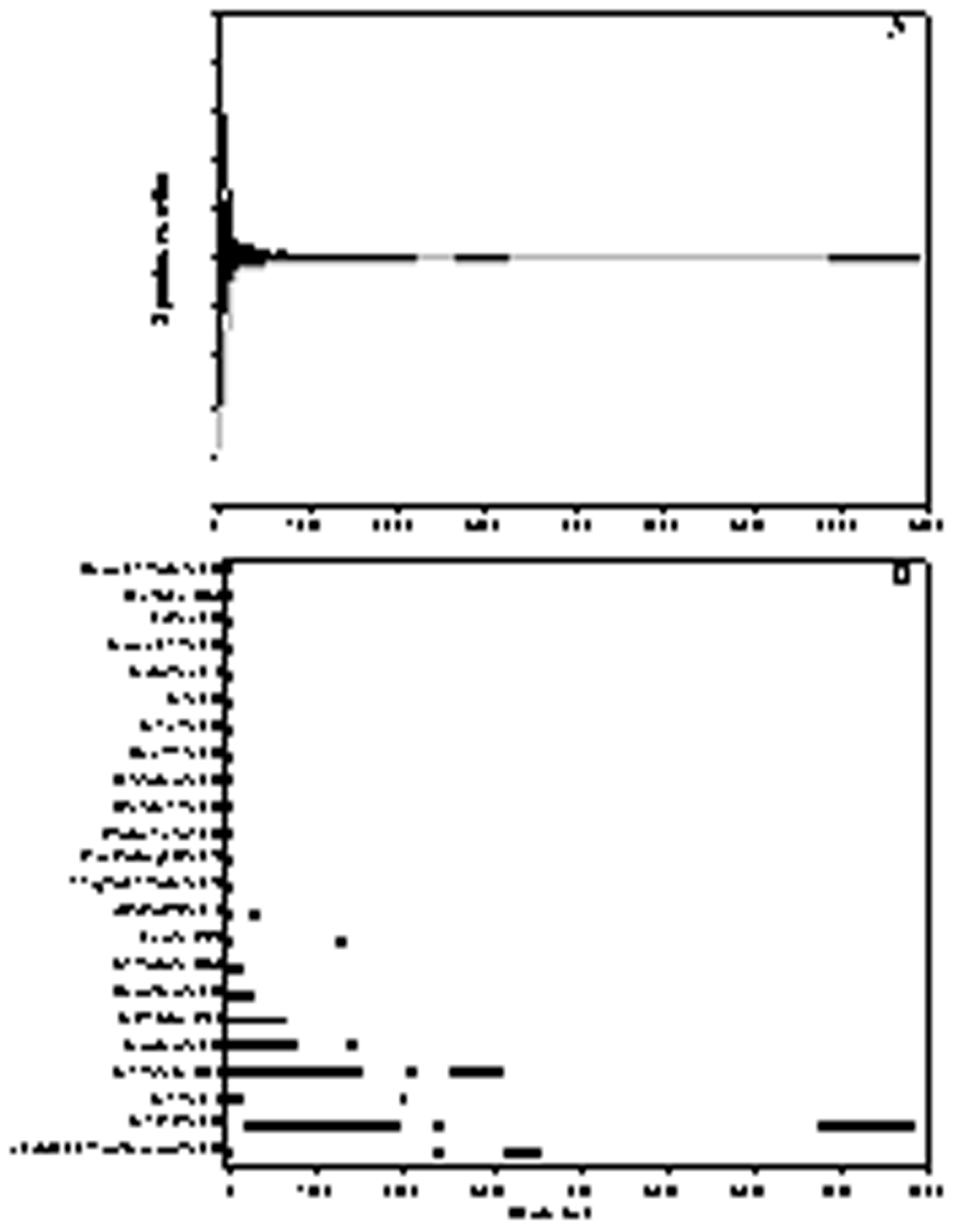
Depth distribution in Janiridae. A) Species numbers per depth. The abscissa divides the total number of species in half, thus the maximum richness is just under 155 species; B) Bathymetric ranges of janirid genera; in brackets are the numbers of species per family

The depth distributions of the 23 genera in the isopod family Janiridae and their 174 species have been analysed as well as the global and depth distributions of the genus *Jaera* itself. The majority of the species within the Janiridae occur in the intertidal and shallow shelf waters within 100 m depth (Fig. 3A), but single species can be found down to abyssal and hadal depths, like *Janthura abyssicola* Wolff 1962 and *J. bougainvillea* (Birstein 1963) (Fig. 3B). Of the 23 genera, 14 have so far solely been recorded within the first 100 m of water depth and only in 5 genera species exceed into the deep sea beyond 1,000 m (Fig 3B). The presently known bathymetric records of *Jaera* species show a 1,300 m depth gap between the 20 previously known species and the new discovered species described herein (Figs. 3B).

### Taxonomy

Family Janiridae Sars 1897

Genus *Jaera* Leach 1814

Full synonymy see Wilson and Wägele [50]: 706

#### Composition

*Jaera albifrons* Leach 1814 type species; *Jaera bocqueti* Veuille et Kocatas in Veuille 1979;*Jaera caspica* Kesselyák 1938*; Jaera forsmani* Bocquet 1950; *Jaera hopeana* Costa 1853; *Jaera ischiosetosa* Forsman 1949; *Jaera italica Kesselyák 1938; Jaera nordica* Lemercier 1958*; Jaera nordmanni* (Rathke 1837)*; Jaera massliensis* Lemercier, 1958; *Jaera petiti Schulz 1953; Jaera posthirsuta Forsman 1949*; Jaera praehirsuta* Forsman 1949; *Jaera sarsi* Valkanov 1936; *Jaera schellenbergi* Kesselyák 1938; *Jaera sorrentina* Verhoeff 1949; *Jaera syei* Bocquet 1950; *Jaera wakishiana Bate 1865; *Jaera tyleri* sp. nov.

* Species status has to be confirmed as description insufficient (pers. comm. A. Rozenberg)

Diagnosis modified after Wilson and Wägele [50], [51]

Body moderately broad, pereonites subequal or becoming broader posteriorly (in males), lateral margins rounded without incisions, coxae not visible in dorsal view. Anterior margin of head without rostrum, slightly convex or nearly straight, labrum protruding between antennae. Eyes dorsal, ranged from well developed to absence. Pleotelson with single notch for uropods insertion with varying depth in different species (not present in *J. hopeana*). Anus inside pleopodal cavity, covered by opercular pleopods. Antenna 1 article 1 flattened, squat, flagellum with 2–3 articles. Antenna 2 articles 1–4 subequal, article 3 without scale, article 5 and 6 longer than articles 1–4; flagellum with more than 20 articles. Mandible molar parallel-sided, distally truncated, palp large, distal article curved and setose. Maxilliped endite longer than wide, palp subequal in width to endite, article 2 broadest, articles 4 and 5 narrow, slender. All pereopods similar, pereopod 1 carpus not enlarged, without spine-like setae. Pereopods 2–7 dactyli with 3 claws (recorded as 2 claws for some species e.g *J. forsmani* and *J. praehirsuta*). Male pleopods 1 and 2 opercular in different degree, pleopod 1 broad, distolateral lobes projected laterally or ventrally, distomedial lobes various, distinct from distolateral lobes. Pleopod 2 stylet subequal in length to protopod, not coiled. Pleopod 3 endopod without plumose setae, exopod longer than endopod, biarticulated with enlarged distal article, male pleopods 3 opercular, covering pleopodal cavity (for the new species and Mediterranean group of species).

Uropods inserted adjacent to each other, at least protopods not protruded behind outline of pleotelson, protopod parallel-sided, squat, much shorter than pleotelson, rami reduced, shorter than protopod.

#### Biogeographic remarks

**Figure 4:**
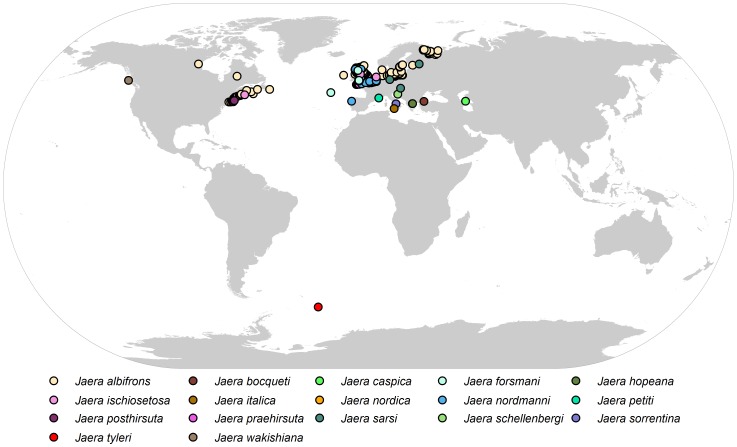
Global distribution of *Jaera*.

In total 678 species-unique georeferenced locations for all species of *Jaera* have been analysed. All species of *Jaera*, apart from the new Antarctic one, are reported from shallow waters and the northern hemisphere (Fig. 4).

*Jaera tyleri* sp. nov. Brandt & Malyutina, 2014

urn:lsid:zoobank.org:act: 2B77D993-22F1-4EB0-906A-7D210E3E0437.

Figs 5–16

**Figure 5:**
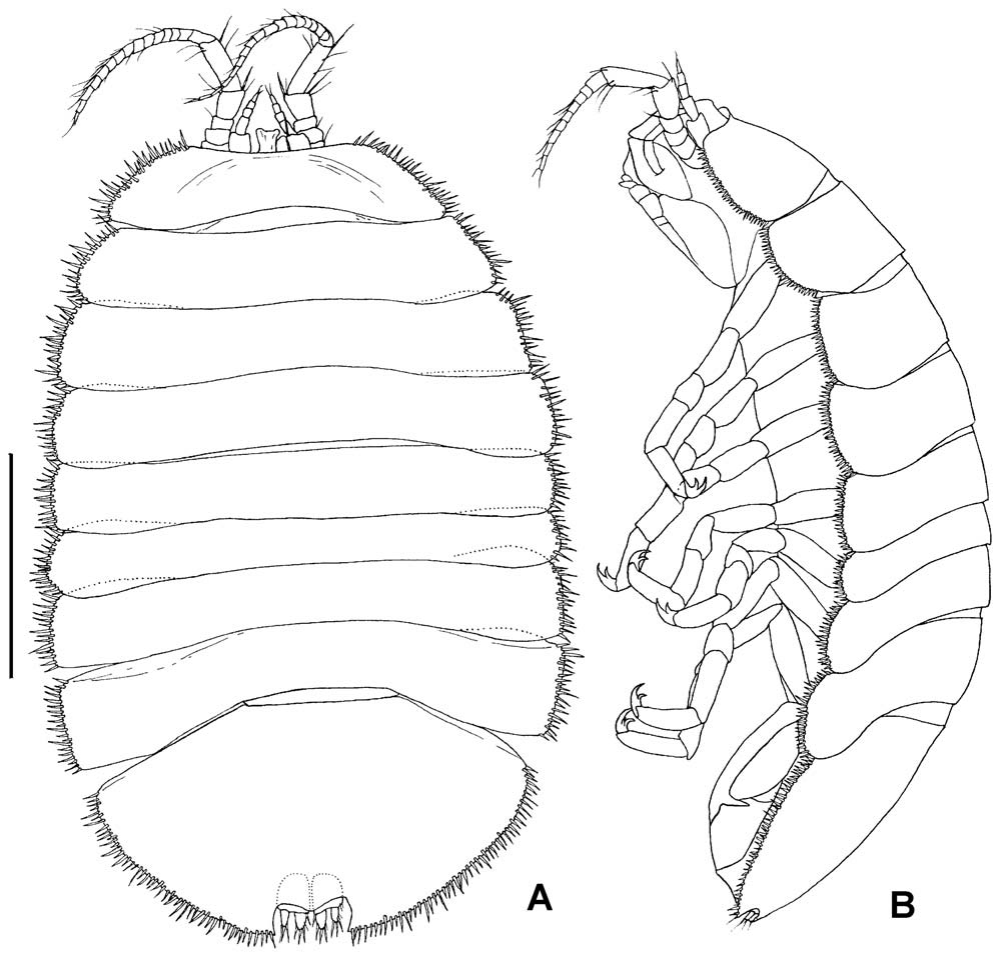
Holotype NHMUK 2014.268, male, total view. A) dorsal view, B) lateral view, scale – 1 mm

**Figure 6:**
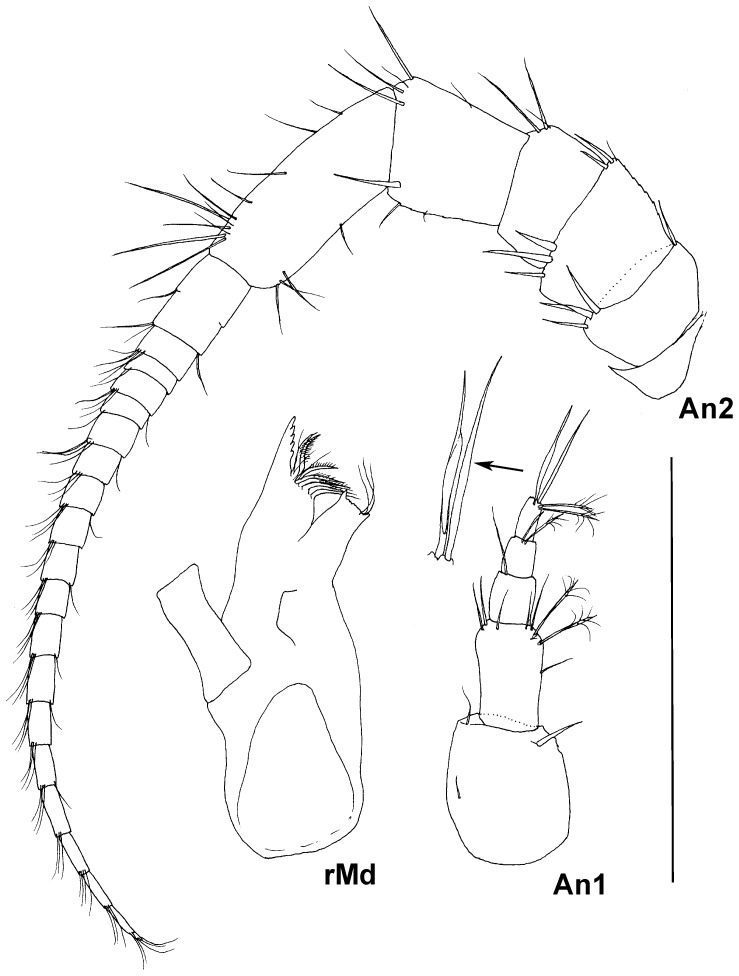
Paratype ZMH K-44090, male, antennula and right mandible, scale – 0.5 mm.

**Figure 7:**
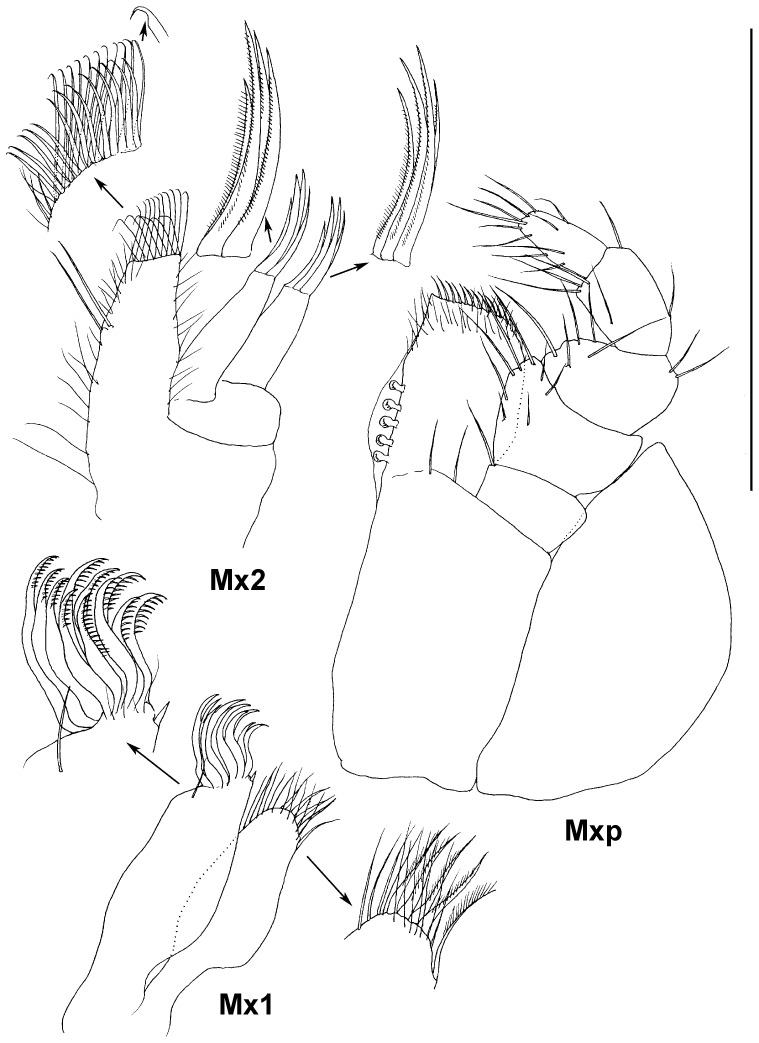
Paratype ZMH K-44090, male, mouth parts, scale – 0.5 mm.

**Figure 8:**
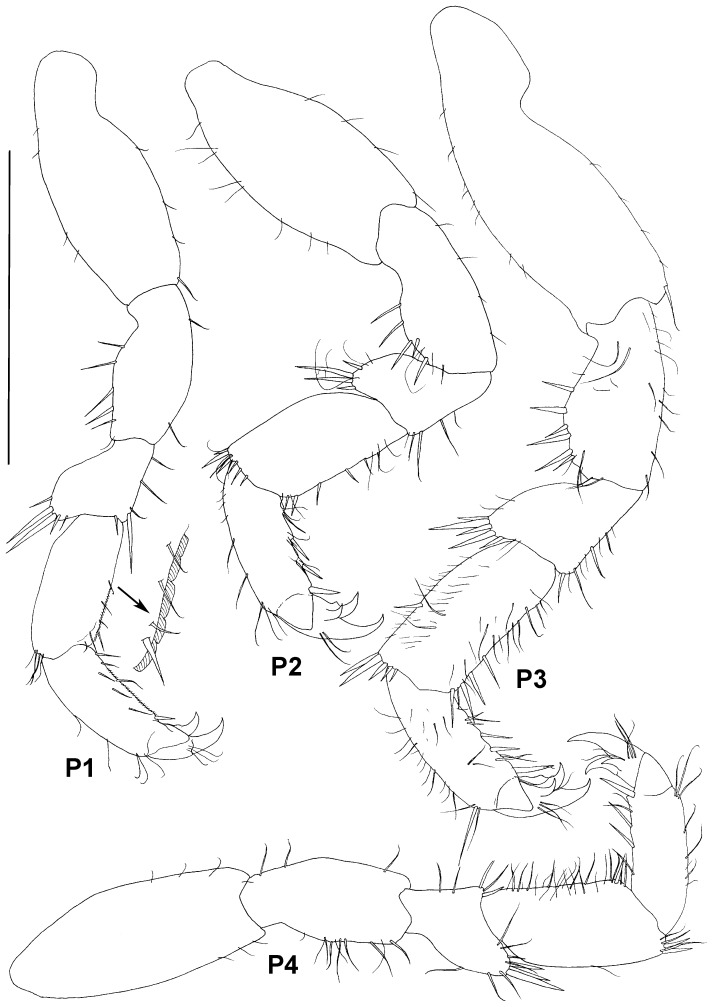
Paratype ZMH K-44090, male, pereopods 1–4, scale – 0.5 mm.

**Figure 9:**
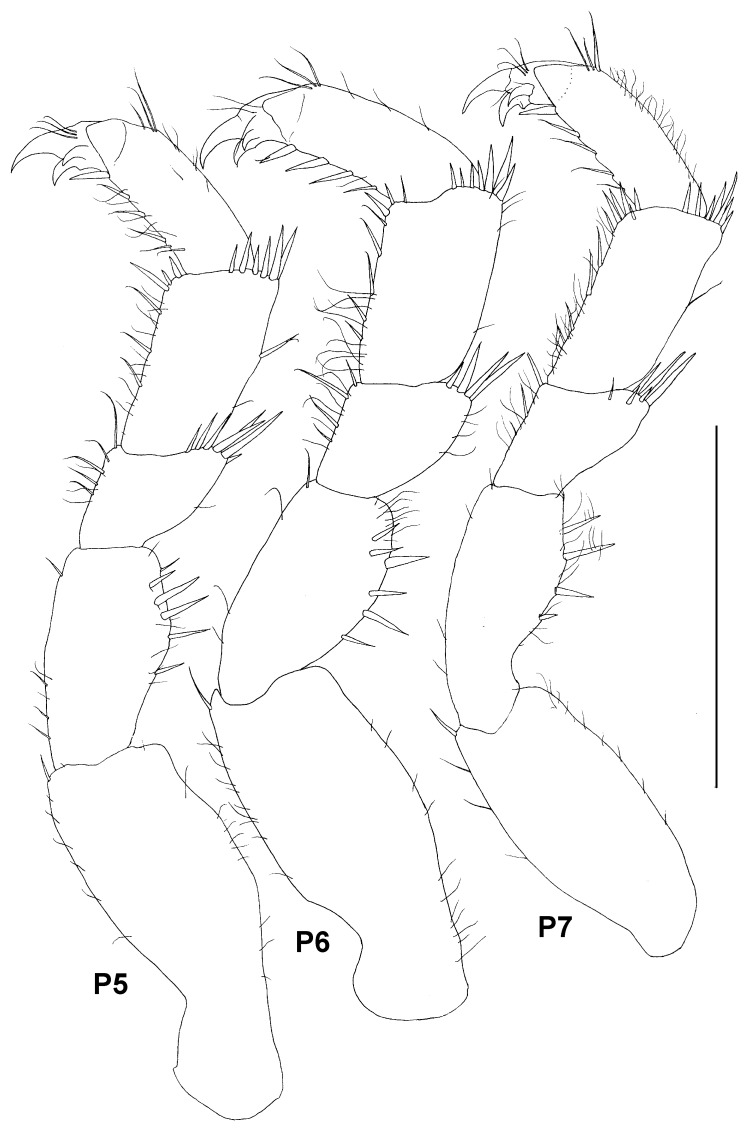
Paratype ZMH K-44090, male, pereopods 5–7, scale – 0.5 mm.

**Figure 10:**
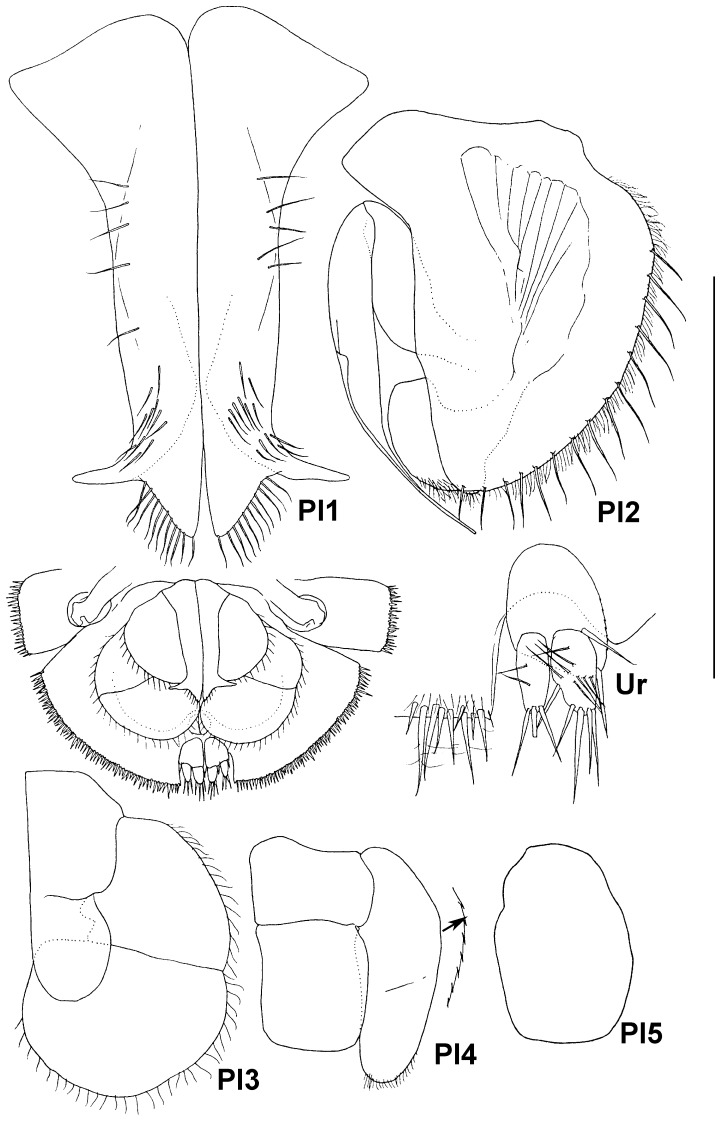
Paratype ZMH K-44090, male, pleotelson, ventral view, pleopods 1–5, uropod, scale – 0.5 mm for pleopods 1 and 2 and uropod and 1 mm for pleopods 3–5.

**Figure 11:**
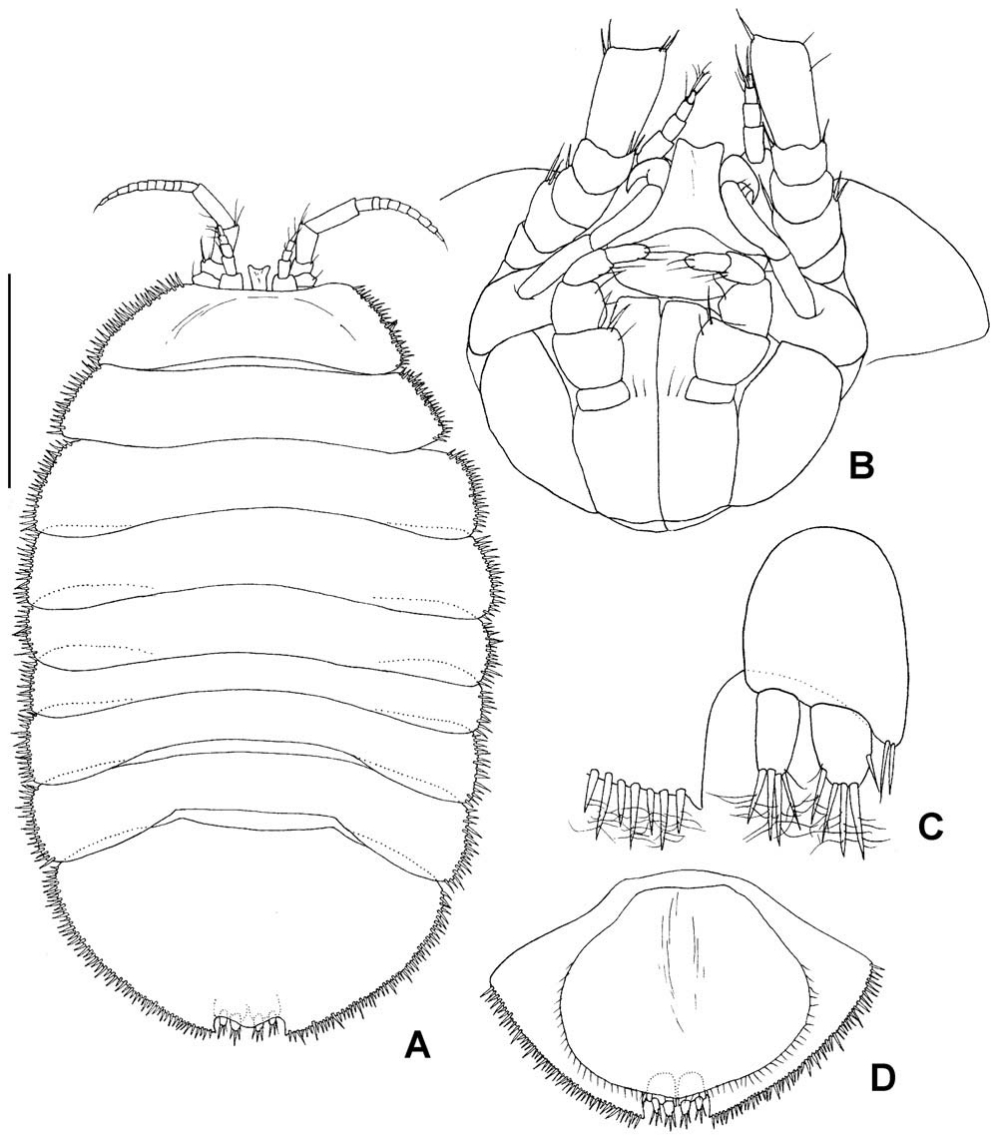
Paratype ZMH K-44089, female. A) dorsal view, B) head, vental view, C) inserted uropod, D) pleotelson, ventral view, scale for total view – 1 mm

**Figure 12:**
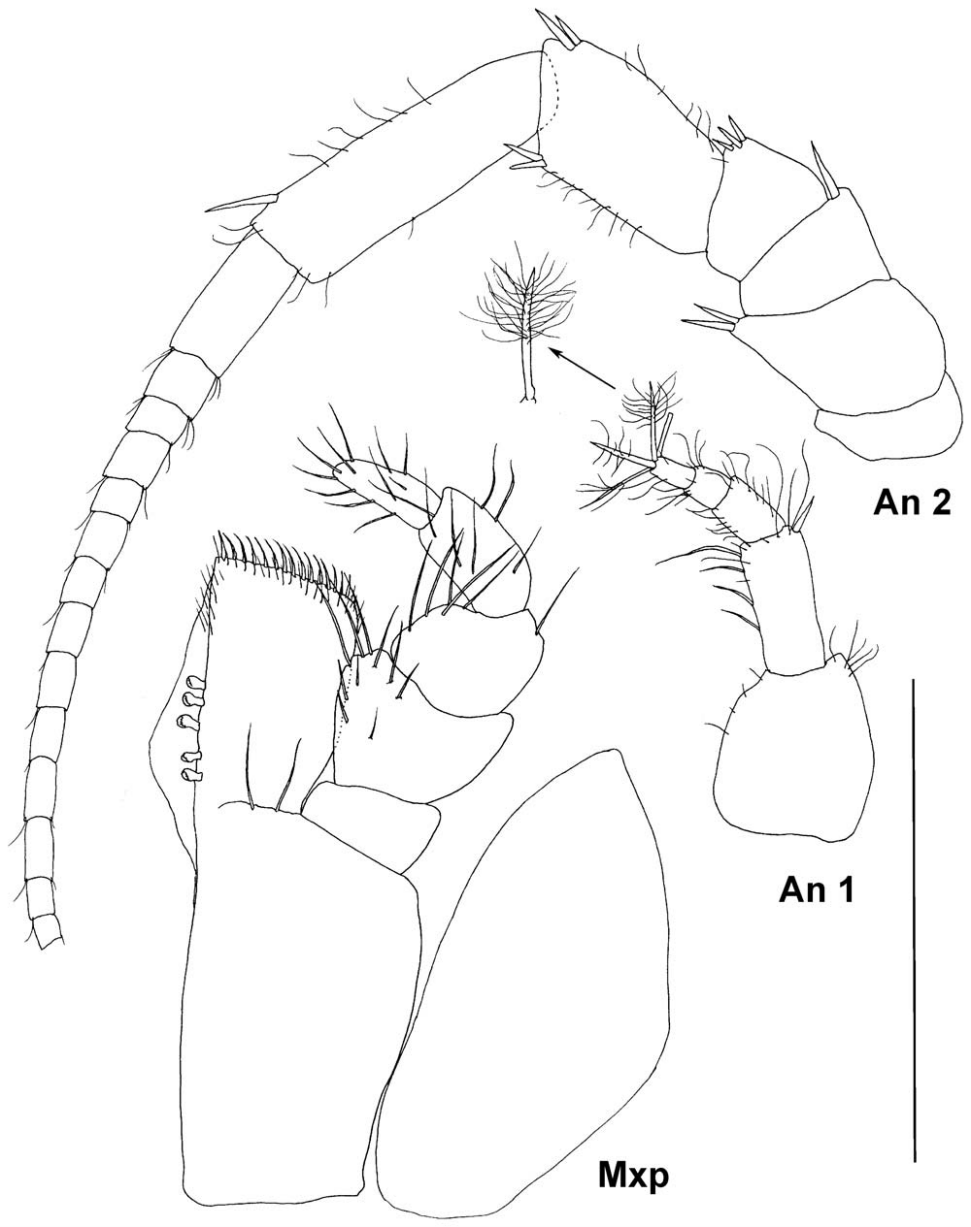
Paratype ZMH K-44089, female, antennula, maxilliped, scale – 0.5 mm.

**Figure 13:**
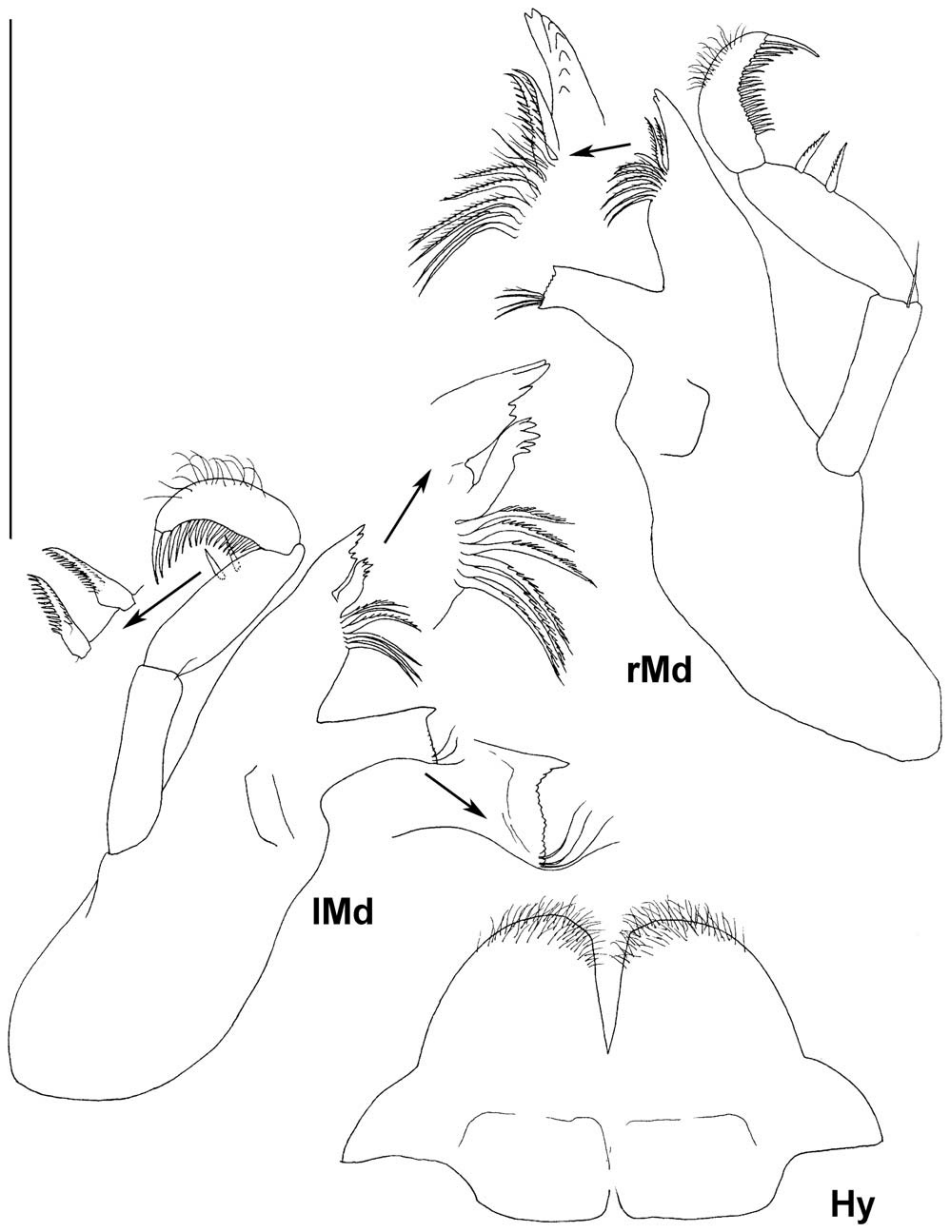
Paratype ZMH K-44089, female, mandibles, hypopharynx, scale – 0.5 mm.

**Figure 14:**
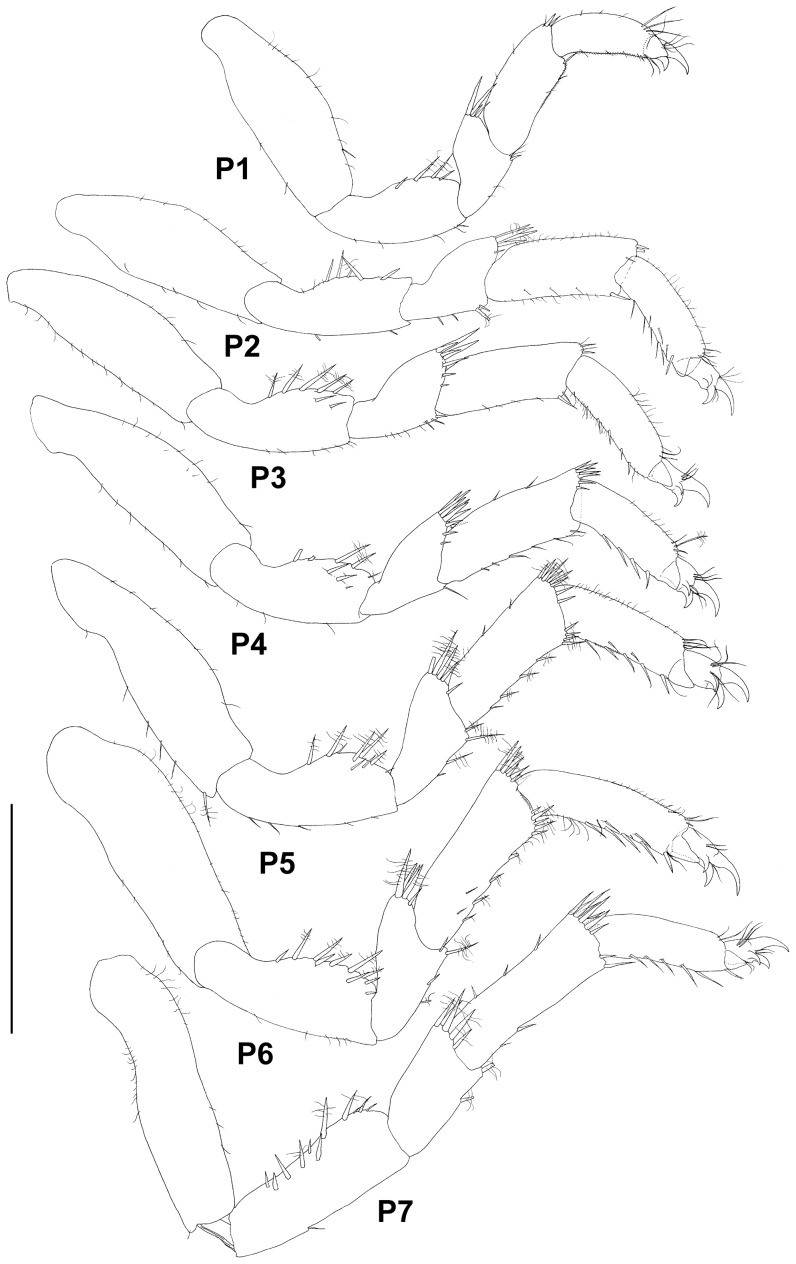
Paratype ZMH K-44089, female, pereopods 1–7, scale – 0.5 mm.

**Figure 15:**
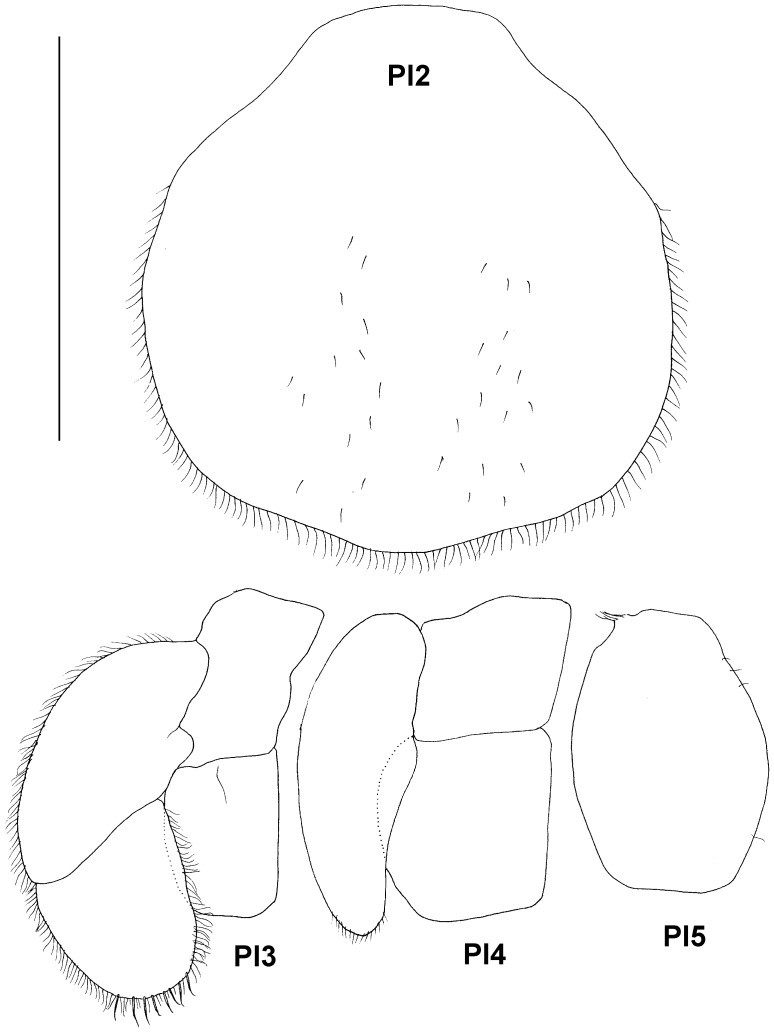
Paratype ZMH K-44089, female, pleopods 2–5, scale – 1 mm.

**Figure 16:**
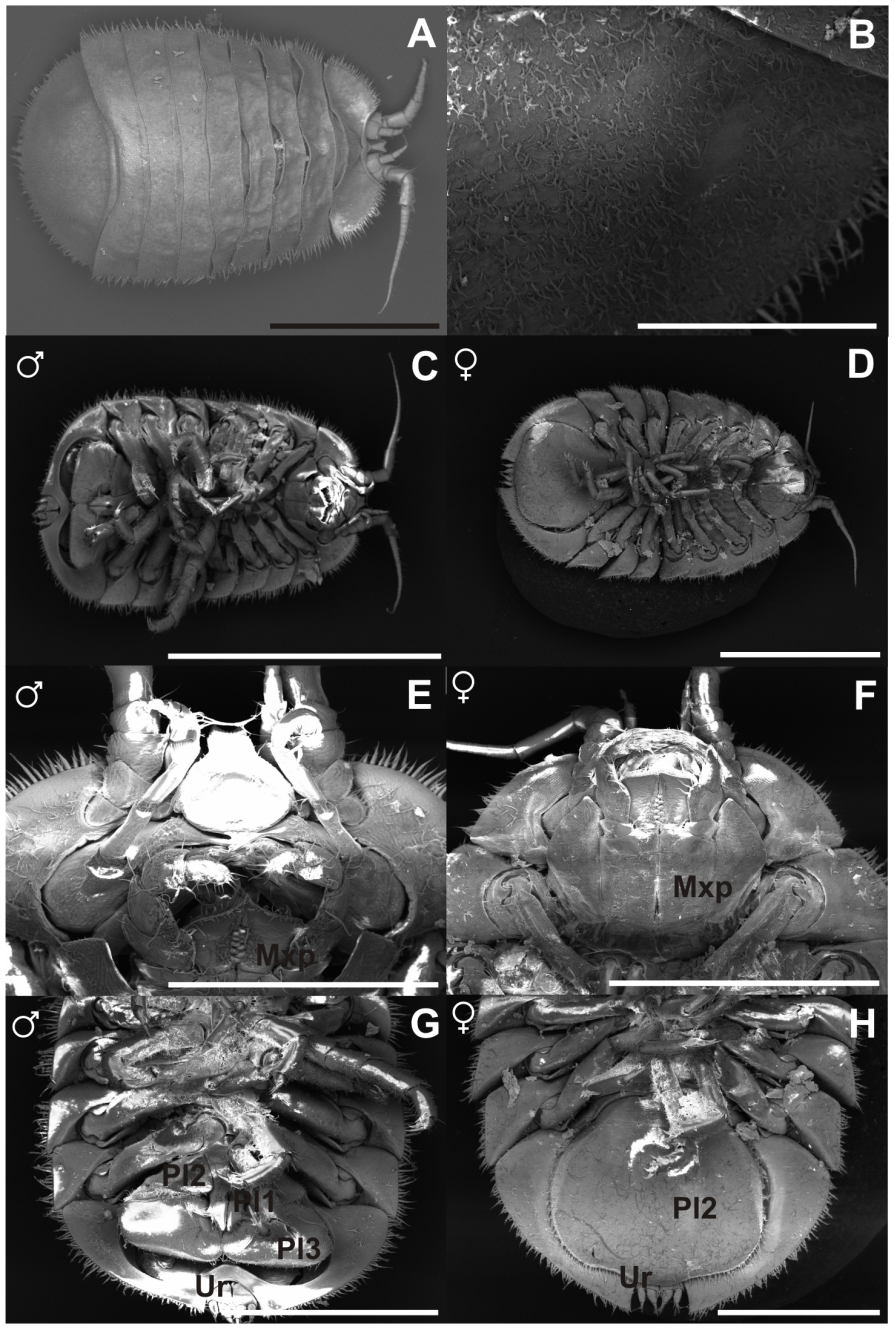
Paratypes NHMUK 2014.340-345, SEM. A) male, dorsal overview, scale – 1 mm, B) male, dorsal setae, scale − 0.3 mm, C) male, ventral overview, scale − 2 mm, D) female, ventral overview, scale − 2 mm, E) male, ventral view, head, maxilliped, scale − 0.5 mm, F) female, ventral overview, head, maxilliped, scale − 1 mm, G) male, ventral view, pleopods 1, 2 and 3, uropod, scale − 1 mm, H) female, ventral view, pleopod 2, uropod, scale − 2 mm

#### Material examined

Whale bones, South Sandwich Islands, Kemp Caldera, RRS *James Cook*.

**Figure 17:**
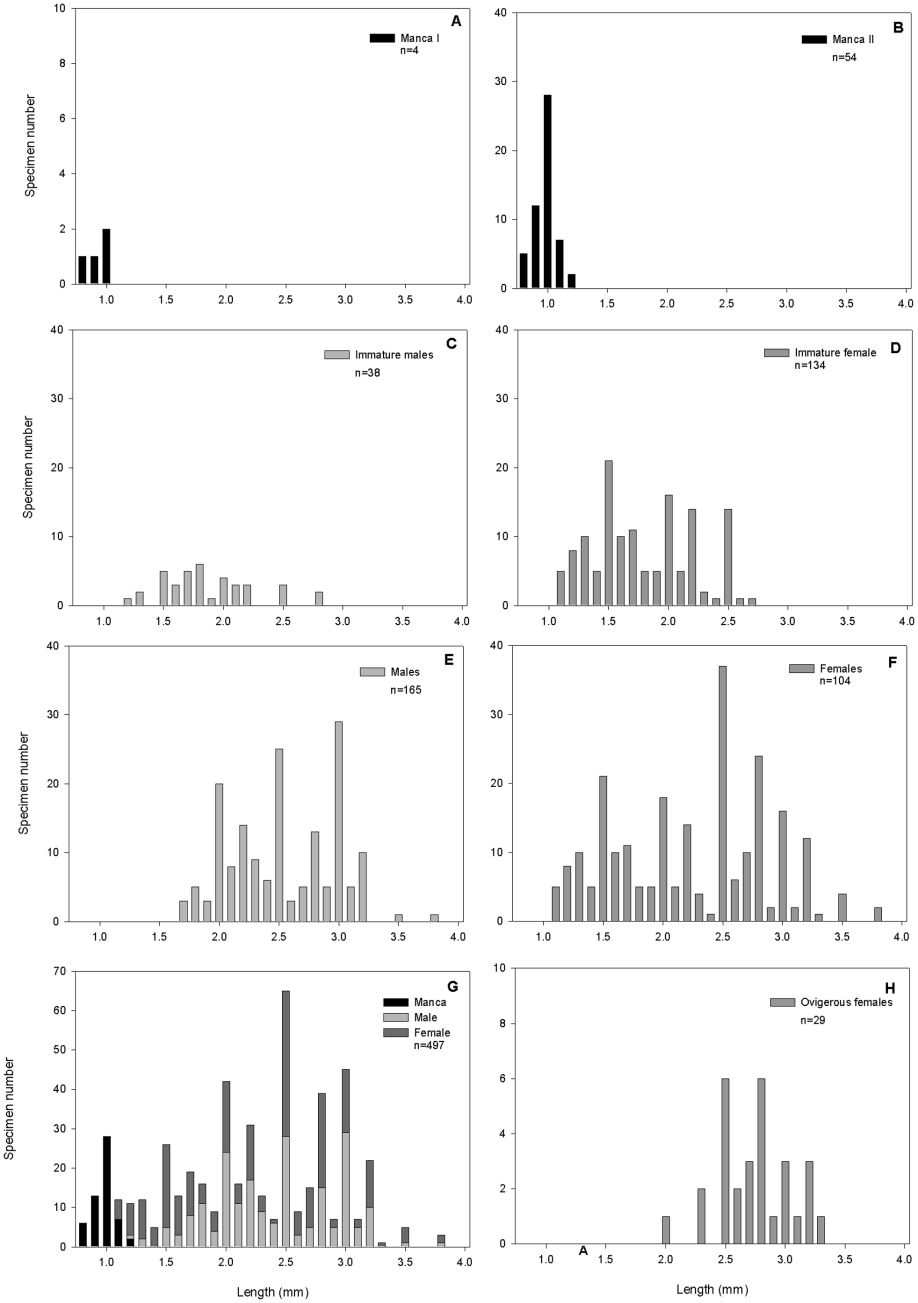
Size-frequency distribution. A) Manca I, B) Manca II, C) immature males), D) immature females, E) males, F) Females, G) all stages joint, F) ovigerous females.

Holotype: NHMUK 2014.268, male (3.6 mm), from whale bone JC42-F-571 Expedition JC42 RRS *James Cook*, Station JC42-5-6 *ISIS* 148, 08.02.2010, 59°41.6 S, 28°21.116 W, 1,445 m (Fig. 5). *Paratypes:* All paratypes were collected during expedition JC42 of RRS *James Cook* at station JC42-5-6 on ROV dive *ISIS* 148, 08.02.2010, 59°41.6 S, 28°21.116 W (DDM), between 1444-1447 m at the same locality (Tab. 1). Type material is deposited at the Natural History Museum, London (NHMUK), the Zoological Museum, Hamburg (ZMH), the British Antarctic Survey (BAS) and the National Oceanographic Centre Southampton (NOCS). The female drawn came from JC42-F-0599 and is deposited as ZMH K-44089 (Fig. 11), the male used for dissection of mouthparts, pereopods and pleopods came from JC42-F-0599 and is deposited as ZMH K-44090 (Fig. 5). The specimens on stubs for the SEM study are deposited as NHMUK 2014.340-348 (Fig. 16). The measured specimens from sample JC42-F-0574 are deposited as NHMUK 2014.269–278 (ovigerous females), NHMUK 2014.279–288 (females), NHMUK 2014.289–298 (immature females), NHMUK 2014.299–308 (males), NHMUK 2014.309–318 (immature males), NHMUK 2014.319–328 (Manca II) and NHMUK 2014.329–338 (Manca I).A total of 1258 specimens have been collected of which 499 were measured and maturity assessed; 4 manca I of 0.8–1.0 mm lengths, 54 manca II of 0.8–1.2 mm lengths, 134 immature females of 1.1–2.7 mm lengths, 38 immature males of 1.2–2.8 mm lengths, 104 females of 2.0–3.8 mm lengths and 165 males of 1.7–3.8 mm lengths (Table 1, Figure 17).

**Table 1:** *Jaera tyleri* sp. nov. Paratype information and collection numbers.

	*Jaera tyleri* sp. nov.										Paratype s
Whale bone	Sample id	N	m	M I	M II	im ♂	♂	im ♀	♀	ovi ♀	Collection numbers
JC42-F-0542, jaw											
	JC42-F-0574	640	343	4	54	27	117	104	23	14	NHMUK 2014.269—348,
											BAS JC42-F-0574
JC42-F-0549, vertebrae											
	JC42-F-0532	56	56			3	21	5	23	4	ZMH K-44093
	JC42-F-0695	38	-								
JC42-F-0571, vertebrae											
	JC42-F-0559	11	11			1		6	4		NHMUK2014.268, ZMH K-44089, ZMH K-44090, ZMH K-44094
	JC42-F-0562	100	-								NOC JC42-F-0562
	JC42-F-0569	218	-								NHMUK 2014.349-358
	JC42-F-0586	10	-								BAS JC42-F-0586
	JC42-F-0587	6	6				2		4		ZMH K-44091
	JC42-F-0603	83	83			7	25	19	21	11	ZMH K-44092
JC42-F-0590, jaw											
	JC42-F-0599	11	-								
	JC42-F-0545	60	-								NOC JC42-F-0545
	JC42-F-0628	25	-								BAS JC42-F-0628
		1258	499	4	54	38	165	134	75	29	

Diagnosis: Head without eyes, frontal margin smooth, slightly concave, antenna 2 length about third of body length, pleotelson posterior notch with sinuate medial projection separating uropods, uropod rami not exserted from the notch behind outline of pleotelson. Male pleopods 1 and 2 smaller than enlarged opercular pleopods. Male pleopods 1 with distolateral lobes elongate, protruded laterally, distomedial lobes triangular, protruded posteriorly, as long and twice as broad as distolateral lobes.

#### Description

Holotype male: body (Fig. 5) length 1.5 widths of pereonites 6 and 7, body height 0.25 body length. Head length 0.2 width; labrum visible dorsally, protruding between proximal article of antennula, distally convex. Pereonites slightly increasing in width posteriorly, lateral margins slightly rounded, longer than medial part of pereonites, posterolateral margins overlaping anterolateral margins of foremost pereonites, coxae not visible in dorsal view. Pereonite 1 length 1.1 head length, width 1.2 head width; pereonites 1–4 and 6-7 almost of same length, pereonite 5 shortest, length 0.6 pereonite 1 length. Pleotelson length 0.6 width, 0.3 body length, as wide as pereonite 1, terminal notch depth 0.15 pleotelson length, medial projection of the notch 0.3 of notch length, uropods rami tips not reaching outline of pleotelson from the notch; body outline beside frontal margin of head and pleotelson notch surrounded with stout setae of alternating lengths (short/long).

Antennula of male (Fig. 6) subequal in length to head; article 1 length 1.1 width, with 1 distomedial stout seta and 2 small lateral setae; article 2 0.8 length and 0.55 width of article 1, with 5 simple and 2 broom setae distally and 1 simple medial seta; article 3 0.45 length of article 2, with 1 distal simple seta; article 4 0.7 length of article 3, with 2 distal broom setae; article 5 1.1 of article 4 length, with 2 long simple and 2 broom setae distally.

Antenna 2 of male (Fig. 6) 0.4 body length, articles 1 shortest and narrowest, articles 2 and 4 of similar size, length about 0.5 width; article 2 with 1 distomedial and 4 distolateral simple stout setae; article 4 with 3 distomedial setae; article 3 1.5 length and 1.1 width of equal articles 2 and 4, with 1 slender and 2 stout distomedial setae and 3 stout distolateral setae; article 5 1.7 length and 0.9 width of article 4, with 1 medial, 4 distomedial, 1 lateral and 2 distolateral simple setae; article 6 1.7 as long and 0.8 as wide as article 5, with 4 lateral simple setae and 11 medial simple setae of varying lengths. Flagellum length 1.2 peduncle length, of 21 articles, articles tapering distally, about 0.3 as long as longest first flagellar article; articles 3-20 with 3 distomedial slender setae, some articles with distolateral seta.

Mandible of male (Fig. 6) incisor process with 5 distally rounded cusps; spine row with 8 spines on right mandible; molar process 1.8 as long and 1.3 as wide as incisor process on proximal part, ventral margin of triturative surface with a few denticles and 3 setae; condyle 0.1 of body length, about half of molar length; palp broken off in male.

Maxilla 1 (Fig. 7) lateral endite 1.1 as wide as mesial endite, distal margin truncated with short spine-like distomedial seta and 11 stout curved comb-like setae, articulated more medially; mesial endite distal margin rounded with tuft of slender setae and 5 stout setulated setae.

Maxilla 2 (Fig. 7) mesial endite broadest, middle endite narrowest, lateral endite slightly wider than middle, all endites of approximately the same length; mesial endite distal margin truncated, with two rows of stout setae ending with small hook; lateral and middle endites each with 4 comb-like distal setae (2 long and 2 slightly shorter).

Maxilliped (Fig. 7) basis length 2.7 width, endite with 5 coupling hooks, two proximal setae ventrally, distal margin straight, with row of slender setae curved medially; palp length 0.9 basis length; article 1 with 1 long simple distomedial seta; article 2 broadest, width 1.3 endite width, medial margin slightly convex, with 5 marginal and 3 ventral simple setae; article 3 0.8 as wide and as long as article 2 laterally, with 2 distolateral setae, medial margin slightly convex, with 5 setae, length 0.65 article 2 length; article 4 as long articles 3 medially, with 3 distomedial setae and 1 lateral seta, width 0.6 article 3 width; article 5 0.7 as long and 0.6 as wide as article 4, with 11 simple setae. Epipod reaching distal margin of palp article 2, twice as long as wide, with rounded lateral margin.

Pereopods subequal in size, shape and setation. Basis longest and broadest article with varying numbers of simple small setae; ischium with dorsal rounded expansion bearing spine-like setae and small simple setae ventrally; merus subtriangular, with distodorsal projection bearing spine-like setae; carpus subrectangular, subequal in width to ischium, with distal spine-like setae (distodorsal setae longer and more numerous); propodus subequal to carpus in length and about 0.7 of carpus width, with stout setae ventrally; carpus and propodus of pereopods 3–7 more setose in comparison to pereopods 1 and 2; dactylus dorsal claw about as long as article, 3 slender dorsal setae proximally of claw, ventral claw about half size of dorsal claw, third claw of pereopods 2–7 (accessory seta) slightly smaller than ventral claw.

Pereopod 1 (Fig. 8) 0.35 times body length, length ratios of ischium-dactylus (together with dorsal claw) to basis: 0.6, 0.3, 0.55, 0.55, 0.3; ischium with 5-6 stout dorsal setae and few simple ventral setae; merus with 1 long and 1 short stout distoventral and 3 ventral setae and 2 long and 3 short stout distodorsal setae; carpus with 4 slender distodorsal setae and 5 simple ventral setae; propodus with 1 distoventral stout seta, 6 ventral simple setae, 2 slender dorsal and 3 distodorsal setae; ventral margin of carpus and propodus fringed with tiny setules; dactylus with 2 claws.

Pereopod 2 (Fig. 8) slightly longer than pereopod 1; length ratios of ischium-dactylus to basis: 0.7, 0.3, 0.55, 0.55, 0.4; carpus width 1.25 pereopod 1 carpus width, armed with stronger setae than in pereopod 1: 6 stout and 3 slender ventral setae and 8 distodorsal setae; propodus with 4 stout and 4 slender ventral setae and 9 dorsal setae.

Pereopod 3 (Fig. 8) length 1.3 pereopod 2 length, proportionally broader and more setose than first pereopods; length ratios of ischium-dactylus to basis: 0.5, 0.3, 0.5, 0.5, 0.3; carpus with 5 stout, 8 slender ventral setae and numerous slender setae dorsally and laterally; propodus with 8 stout ventral setae and numerous dorsal and some lateral slender setae.

Pereopod 4 (Fig.8) as long as pereopod 2, shorter than pereopod 3, length ratios of ischium-dactylus to basis: 0.65, 0.3, 0.7, 0.6, 0.4; dorsal setae on ischium and setae on carpus more slender than on pereopods 2–7; merus with 5 simple distoventral setae, distodorsal projection with 3 stout and 7 slender setae; carpus with 5 distodorsal setae, 1 dorsal and many ventral simple setae; propodus with 6 ventral and 8 dorsal setae.

Pereopods 5–7 (Figs. 9) subequal in shape and size, pereopod 7 slightly narrower than others; for all three pereopods length ratios of ischium-dactylus to basis approximately: 0.6, 0.3, 0.5, 0.5, 0.3; ischium with 5–6 stout and few slender dorsal setae; merus with 5–7 stout distodorsal setae and 8 ventral setae; carpus with 6–7 distodorsal and 1–4 dorsal setae and 6–8 stout and many simple ventral setae; propodus with 4–5 stout ventral setae, 3 distodorsal and some slender dorsal setae.

Pleopods of male (Fig. 10): pleopod 1 length 1.45 proximal width, and 3.1 midlength width; each side of ventral surface with row of 5 setae and groups of 11–13 setae proximally of distolateral lobes; the distolateral lobes slender, projected laterally, medial lobes triangular, subequal in length and twice as broad as lateral lobes, distolateral margin of each medial lobe with 11 setae. Pleopod 2 protopod length 1.1 width, 0.7 pleopod 1 length, laterally fringed with long submarginal setae and short bristles in between, stylet of endopod 0.9 of protopod length, slender and acute distally, exopod thickened, prominent behind distomedial margin of protopod, with tuft of fine setae. Pleopod 3 opercular, 1.7 of pleopod 2 length; protopod elongate, length 1.4 width, endopod small, rounded, without any setae, slightly shorter and narrower than protopod, exopod 1.4 as long as wide, 2.6 as long and as wide as endopod, laterally and distally fringed with short simple setae, distal article as long and 1.35 as wide as proximal article. Pleopod 4 protopod length 0.75 width, endopod subquadrangular, visibly larger than endopod of pleopod 3, length 1.15 width, exopod 2.5 as wide and 1.9 as long as endopod, with short row of distal setulae. Pleopod 5 one lobe, length 1.55 widths.

Uropod (Fig. 10) of male length 0.25 pleotelson length; protopod length 1.35 width, with 2 long setae on small distomedial projection, endopod 0.9 length and 0.45 width of protopod, with 7 ventral and 5 long distal setae; exopod 0.9 length and 0.75 width of endopod, with 3 distal and 2 ventral setae.

Additional description of female (Figs 11–15). Proportions of habitus and body parts in females are rather equal to those of male; differences will be described in the following.

Body (Fig. 11) length 1.6 pereonite 3 width, pereonites 3–7 of same width, not broadened posteriorly like in male, caudal notch of pleotelson shorter than in male, 0.1 of pleotelson length.

Antennae (Fig. 12) more slender, antenna 2 less setated than in male. Antennular article 1 length 1.2 width, with 4 lateral and 5 distomedial fine setae, article 2 0.8 as long and 0.4 as wide as article 1, with 7 lateral setae, 1 stout and 3 long slender distomedial setae; articles 3–5 evenly tapering distally, with many fine setae, article 3 0.5 as long as article 2, articles 4 and 5 0.5 as long as article 3, article 5 with 4 long distal broom setae. Antenna 2 0.3 of body length, article 2 broadest (in male article 3 broadest), distolateral projection with 2 stout setae, articles 3–6 more slender than in male, articles 3 and 4 subequal in size, about 0.8 as long and as wide as article 2, article 3 with 1 distomedial stout seta, article 4 with 3 distomedial stout short setae, article 5 twice as long as article 4 medially, with 4 distal stout setae and many simple short setae, article 6 1.5 as long as article 5 with 1 stout distomedial seta and several simple slender setae. Flagellar article 1 longest, 0.4 as long as article 6, with only 2 (proximally) and 1 (distally) simple setae.

Mandibles (Fig. 13) with 3 long and 2 short teeth on left incisor (8 on right mandible), lacinia mobilis of left mandible slightly bent distally, with 5 teeth, 0.7 length of incisor process; spine row with 6 spines on left and 8 on right mandibles respectively; molar process 0.8 length and 0.6 width of incisor process on proximal part, triturative surface concave, with long dorsal and many lateral denticles and 3 ventral setae; condyle 0.1 times body length, 0.7 length of molar; palp 0.9 body length; article 1 with 1 slender distal seta; article 2 1.1 of length and 1.3 of width of article 1, with 2 bilaterally comb-like setae in distomedial half; article 3 slightly curved, 0.7 as long and as wide as article 2, with row of stout ventral setae and many fine setae distodorsally.

Hypopharynx (Fig. 13) outer lobes 1.4 of inner lobes width, with dense distal fine setae, inner lobes without setae.

Maxilliped (Fig. 12) as in male.

Pereopods (Fig. 14) of similar shape and proportions, getting longer posteriorly. Pereopods more slender in comparison with male's pereopods (for example, length/width ratios of carpus of pereopods 2, 4 and 6 are 2.6, 2.5 and 3 respectively, for male it is 1.8, 2 and 1.8). Spine-like stout setae on ischium-carpus in female more setulated, but articles have less slender setae. Female pereopod 4 not shorter than pereopod 3 as in male.

Pleopod 2 (Figs 11, 15) about as long as wide, rounded, with shallow medial keel, many simple setae mediolaterally and distally, fine short setae scattered on surface. Pleopod 3 (Fig. 15) different than in male: endopod subrectangular, almost as long and as wide as protopod, exopod 2.35 as long as wide, 2.25 as long and 1.4 as wide as endopod, surrounded with row of fine setae, distally with 8 longer and stouter setae. Pleopods 4 and 5 (Fig. 15) like in male.

Uropod (Fig. 11) length 0.2 pleotelson length; protopod length 1.25 width, with 2 stout medial setae, rami shorter than in male: endopod 0.4 as long and as wide as protopod, with 5 stout setulated distal setae; exopod as long and 0.7 as wide as endopod, with 4 stout setulated setae distally and 1–4 slender simple setae.

Distribution: Southern Ocean, inside Kemp Caldera at depths of 1,444 –1,447 m.

Etymology: The epithet *tyleri* (Latin, masc.) has been given in honour of Prof. Paul Tyler (NOC) in recognition for his continual efforts in deep-sea research and for his leadership of the ChEsSO consortium.

Remarks: *Jaera tyleri* sp. nov. is the only blind species of *Jaera* that has ever been described and can therefore easily be distinguished from other species of the genus by this character as well as by the having the short antenna 2 (about a third of body length) and other characters mentioned in the presented diagnosis. By having small male pleopods 1 and 2 and enlarged opercular pleopods 3 *J. tyleri* is close to a group of Mediterranean species, especially to *J. nordmanni*, which possesses reduced eyes. Total views and details of *J. tyleri* are also provided in SEM images in Fig. 16.

#### Ecological remarks

Specimens of *Jaera tyleri* sp. nov. are found on all sites of the whale bones with exception of the parts that were submerged in the sediment (Fig. 2A, B). The isopods were tightly holding on to the whale bone (Fig. 2C, D), having their pereopods actively stuck into the bone tissue (Fig. 2E, F). Population densities based on the number of specimens sampled per individual collected whale bone are estimated between 470 – 6,000 specimens per m^2^.

In total 499 specimens were sex identified and measured, ranging from 0.8–3.8 mm in length (Fig. 17). Of these 58 specimens were in juvenile manca stages, 203 specimens were males and 238 specimens were females. The observed sex ratio showed a slight bias towards females (1:1.17 males: females). Size-frequency distributions were analysed for stages and sexes of *Jaera tyleri* sp. nov. (Fig. 17), indicating a single cohort in the manca II stage (Fig. 8B) but multiple cohorts in immature females, males and females (Fig. 17D-G).

**Figure 18:**
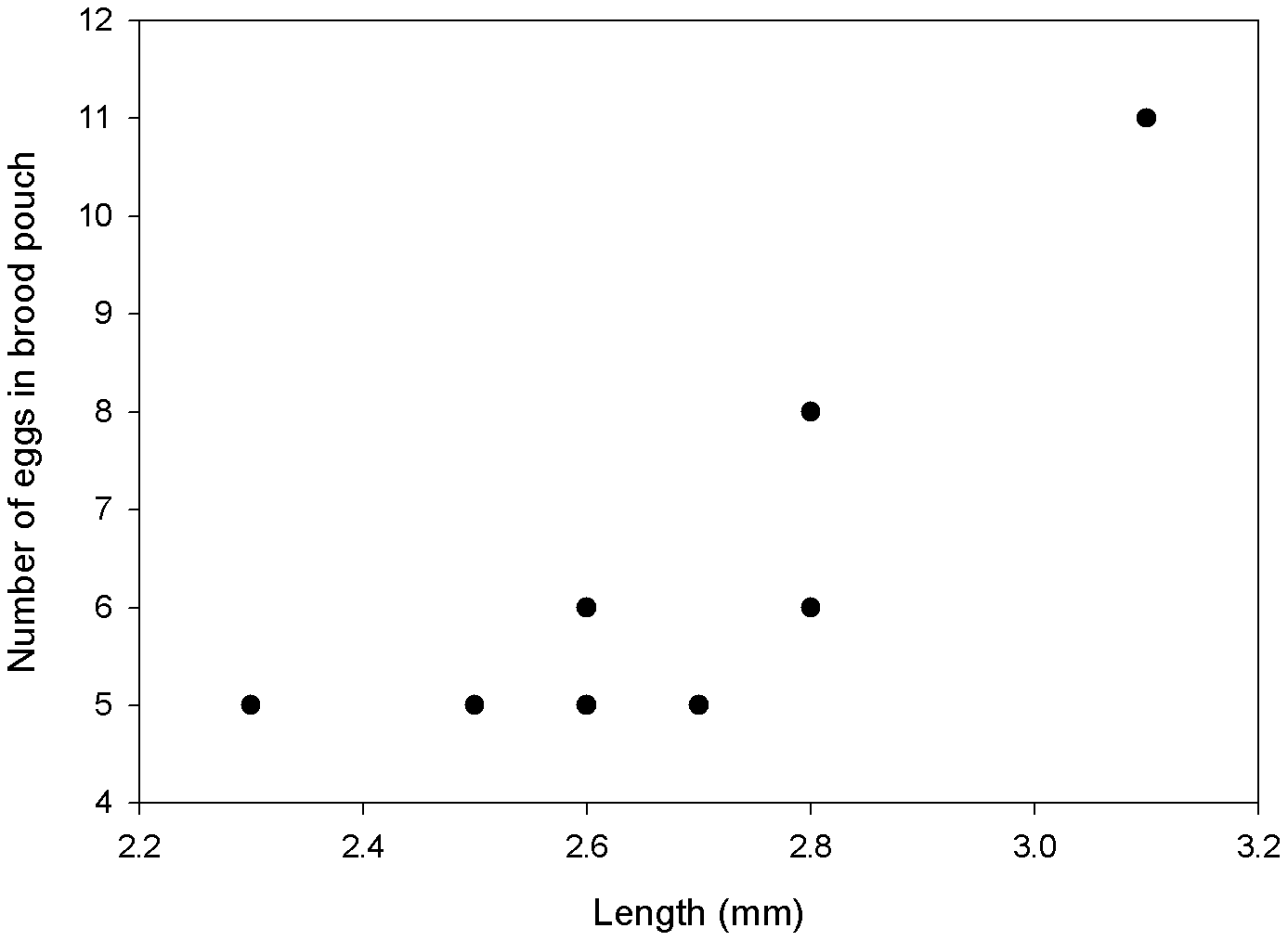
Number of eggs per ovigerous female.

Twenty nine of the 104 mature females were ovigerous, carrying either eggs or juveniles (two specimens of 2.7 and 3.3. mm length) in their brood pouch (Fig. 17H). Their size-frequency distribution indicates that there are multiple reproducing cohorts (Fig. 17H). The numbers of eggs in the brood pouch varied from five to 11 and increases with size of the female (Fig. 18).

### Molecular analysis

The sequencing was successful for 641 bp of *18*S fragments for the specimens JC42F-0574-4 (GenBank KJ174500) and JC42-F-0574-6 (Genbank KJ174501). Both sequences were genetically identical. Sequence alignment with other Isopoda yielded full and truncated datasets of 2,302 and 680 bp respectively, containing 110 taxa. After RNA Salsa analysis, 18.7% and 13.8% of the two respective alignments contained gaps and undetermined characters. Estimated alpha  =  0.22 within the loop regions for 678 bp, indicating a high degree of rate heterogeneity across this partition. For both alignments, the best model according to AIC scores was 6A.

**Figure 19:**
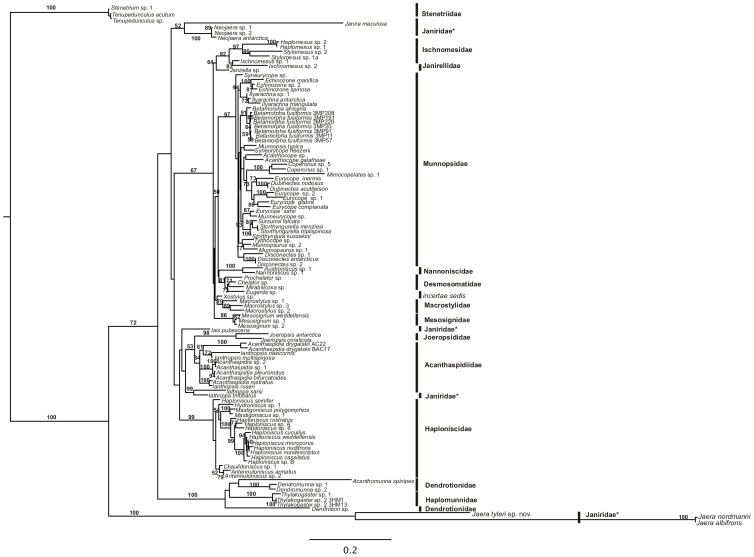
The tree from the RaXML secondary structure model 16A analysis of partial 18S rDNA (2,302 bp). The tree is rooted on the Stenetriidae. The numbers represent bootstrap support with 100 replicates.

The maximum likelihood phylogeny for secondary structure model 6A places *J. tyleri* basal to the two previously published *Jaera* species in a monophyletic group, with 100% bootstrap support from the 2,302 bp alignment (Figure 19) and 86% from the 680 bp alignment (Figure S1). The whole clade is placed basal within the Asellota, although bootstrap support for this position within the phylogeny is very weak (72% for the 2,302 bp alignment). In the 680 bp alignment, *Janira maculosa* is placed basal to the divergent *Jaera* clade but with weak (42%) support. In both phylogenies the Janiridae fall into four clades polyphyletic within the Asellota (*Neojaera*, *Iais*, *Iathrippa* and *Jaera*), but in each case the placement of these clades in the phylogeny is not strongly supported. Earlier analyses of these same genera using more sites (18S and 28S) showed the same pattern [16]. Both phylogenies provide strong support for the *Jaera* species as a highly distinct and evolutionarily divergent clade.

## Discussion

### Taxonomic implications

The main difficulty in identifying the new species was the unusual habitat and the absence of eyes of the species. The new species has the usual shape of *Jaera*, but is blind. All known species of *Jaera* occur in shallow waters of the Northern Hemisphere, mainly the Atlantic and the Mediterranean and bear eyes. Besides a few characters, mainly the reduction of eyes and the short antenna 2, which distinguish the new species from other *Jaera,* the new species possesses a long list of characters (Table 2) which attributes it to *Jaera.* We therefore placing it into this genus what is also supported by the molecular analysis. Species of *Jaera* show different states of eye reduction, which is possibly connected with different life styles: from well developed eyes with 36 ommatidia in common littoral species like *J. albifrons* to reduced eyes with 5–6 ommatidia in *J. hopeana,* an ectocommensal living between the legs of *Sphaeroma serratum.* Species of the morphologically similar genus *Iais* Bovallius 1886 (Table 2), which are ectocommensals of isopods of the genera *Sphaeroma, Exosphaeroma* and other sphaeromatids have small eyes reduced to 2 ommatidia. By the reduction of eyes and by the short antenna 2 *Jaera tyleri* sp. nov. has similarities with some species of the janirid genus *Caecijaera* Menzies 1951, *e.g. C. horvathi* Menzies 1951 (type species), *C. kussakini* Malyutina 1994 and *C cojimarensis* Ortiz et Laalana 1993 (Table 2). *Caecijaera* are commensals of the wood-boring isopod genus *Limnoria* Leach 1814 living in their burrows; however, states of other characters, especially pleopod and antenna 2 morphology, are different to those in *Jaera*.

**Table 2:** Diagnostic character table for *Jaera tyleri* sp. nov.

Genus	*Jaera*	*Caecijaera*	*Iais*	*Jaera tyleri* sp. nov.
Body shape	flattened	flattened	flattened	flattened
Frontal margin of head	Rounded, protruded between antennae	Projected, but not separating antennae	Rounded, protruded between antennae	Almost straight, slightly concave
Lateral margins of pereonites	Entire, broad, rounded	Entire, broad, rounded	Sinuate with posterolateral notch	Entire, broad, rounded
Pleotelson posterior notches	Single notch of different depth not separated medially or with very weak medial projection	Two notches separated by medial projection	Posteriomedial margin projected between uropods	Single notch Separated medially by short medial projection
Eyes	present	absent	present	absent
Coxae	Not visible	Not visible	Visible on all pereonites	Not visible
An1 insertion	Separated by labrum	Inserting adjacent to each other	Separated by head frontal projection	Separated by labrum
An1 article 1	Without distomedial lobe	With distomedial lobe	Without distomedial lobe	Without distomedial lobe
An2 length	About a half of body length or longer	Less than one third of body length	More than a half of body length	About a third of body length
An2 scale	absent	Present, well developed	absent	absent
Article 1 of An2	Small triangular, not visible dorsally	Enlarged, visible dorsally, reaching lateral margin of head	Small triangular, not visible dorsally	Small triangular, not visible dorsally
Dactyli of P2–7	With 3 claws, (illustrated as 2 claws for a few species)	With 2 claws	With 3 claws	With 3 claws
Male Pl1, 2	Some species have Pl1 and 2 smaller than Pl 3, not opercular (plesiomorphy)	Opercular, both together close branchial cavity as female Pl2	Opercular	smaller than Pl 3, not opercular
Distomedial lobes of male Pl 1	Some species have triangular lobes, but in this case lateral lobes not projecting laterally	Not projected	Similar to lateral lobes, both slightly projected posteriorly	triangular lobes, as long as projected lateral lobes
Male Pl 2 stylet	About as long as protopod	shorter than protopod or longer, coiled	Half as long as protopod	About as long as protopod
Male Pl 2 exopod	Swollen	Small, slender	Small, slender	swollen
Male Pl 3	Opercular in SOME species	Closed by pleopods 1,2	Closed by pleopods 1,2	Opercular
Endopod of Pl 3	Without distal plumose setae, narrower and shorter than exopod	With distal plumose setae, broader than exopod	Without distal plumose setae, broader and slightly shorter than exopod	Without distal plumose setae narrower and shorter than exopod
Exopod of Pl 3	Equal or broader and longer than endopod, distal article equal or larger than proximal one	Narrower than endopod, distal article smaller than proximal one	Narrower than endopod, distal article smaller than proximal one	Broader and longer than endopod, distal article larger than proximal one
Uropod	Much shorter than pleotelson; inserting adjacent to each other	Much shorter than pleotelson; separated by medial projection of the Plt	About a half of pleotelson length; separated by medial projection of the Plt	Much shorter than pleotelson; inserting adjacent to each other
Ur protopod	Longer than broad	Broader than long, with medial lobe	Longer than broad	Longer than broad
Ur rami	Shorter than protopod	Subequal to protopod in length	Longer than protopod	Shorter than protopod

The discovery of *Jaera tyleri* sp. nov. and the generic assignment of the janirid species found at southern Californian whale falls to *cf. Janiura* and an unidentifiable but not *Jaera* provides evidence for an underestimated species richness the family Janiridae especially in the deep sea. This study also indicates that the speciose family Janiridae urgently needs to be revised taxonomically, as both morphological and molecular characters, have shown that Janiridae are polyphyletic [16].

### Phylogenetic position of *Jaera tyleri* sp.nov

The isopod *Jaera tyleri* sp.nov. has been characterised with a highly divergent sequence of partial 18S rDNA. This species is challenging to align with other janirids but through careful alignment of this species with other janirids with reference to rDNA secondary structure, maximum likelihood cluster this species with significant (>95%) bootstrap support in a monophyletic group with other members of the *Jaera* genus. Raupach [personal comm.] previously reported extremely divergent 18S rDNA sequences of *Jaera albifons* and *J. nordmanii,* which were subject to long-branch attraction and therefore excluded from his Raupach et al. (2009) dataset [16]. Here we used the 18S rDNA Raupach et al. [16] dataset and included the sequences of *Jaera albifons* and *J. nordmanii* to analyse the phylogenetic position of *Jaera tyleri* sp.nov. Morphological characters support placement of *Jaera* within the Janiridae. The *Jaera* genus is however placed basal to other asellote isopods, with 72% support for this phylogenetic position. It is therefore still unclear where *Jaera* is placed phylogenetically in relation to other isopods. Further taxonomic and molecular survey of this unusual group is warranted to shed more light on this issue. Previous morphological and molecular phylogenetic studies on Isopoda including Janiridae showed evidence that this family is not monophyletic [16], [17] and requires taxonomic revision.

### Biogeography

The isopod family Janiridae is found in all oceans and seas from the intertidal to the hadal, although their distribution is predominantly in shallow waters and on the shelf [15]. Until the discovery of *Jaera tyleri* sp. nov., only four (*Neojaera*, *Janiralata*, *Janira* and *Janthura*) of the 23 janirid genera have been reported from the deep sea, including a record of *Neojaera* from 1,500 m in the Southern Ocean (SO) [52]. Of the 174 globally known janirid species, eleven species of six genera have been recorded from the SO [53]. The genera *Austrofilus* and *Neojaera* are most species rich with three of their species occurring in the SO; the fourth species of *Austrofilus* is known from Spain while the eight species of *Neojaera* show a Gondwanan distribution. *Iathrippa* is present with two species, while species of *Caecianiropsis*, *Ianiropsis*, *Jaera* are singletons. Here their overall bathymetric distribution ranges from the intertidal to 1,524 m, with more species living on the shelf than on the bathyal slopes and abyssal plains [52], [53]. In their analysis of isopod abundances on SO slope and deep-sea Kaiser et al. [54] assessed the Janiridae to be patchily distributed.

The janirid genus *Jaera* has a northern hemisphere centric distribution and the presented new species *J. tyleri* sp. nov. is the first confirmed record of this genus for the southern hemisphere. The latitudinal as well as the bathymetric range of the genus has been extended.

### Isopoda on whale falls

Detailed studies and identification of isopods associated with whale skeletons have been sparse in the past. The munnopsid *Ilyarachna profunda* and its abundance have been recorded from *sulpophilic* stage of whale falls [6]. The latter authors also reported unidentified Janiridae on whale skeletons in southern California. Their isopod collections from vertebrae picks and boxwashes of sampling since 1995 comprise seven morphospecies, now available to us for further identification and comparison with the Antarctic *Jaera tyleri* sp. nov. [Smith unpublished data]. No information is given on abundance of these morphospecies. The taxonomic identification revealed two species of Gnathiidae, two species of *Munneurycope*, one species similar to *Acanthocope* (both genera are Munnopsidae) and two species of Janiridae of which one is similar to the deep-sea genus *Janthura* and the other janirid was unidentifiable to genus. Amon et al. [8], describe the position, age and associated fauna of the Kemp Caldera whale fall, and remark on the presence of a *Jaera* sp. nov. and its occurrences within the whale skeleton, which relates to the species described herein. Their review on the Isopoda associated with whale falls names the Munnopsidae (4 species) and Janiridae (3 species) as the most frequently found and diverse families. While the Munnopsidae are very species-rich and commonly found in the deep sea, especially in the SO (reported with 219 species from the ANDEEP expeditions [55]), the Janiridae are less diverse and predominately shallow water taxa [53]. Gnathiidae are known to be parasitic, blood-sucking parasites on fish [56] and their occurrence near whale falls might be more related to the presence of scavenging fish.

### Ecology

The isopod *Jaera tyleri* sp. nov. occurred in high abundance on whale bones and was absent from non-whale bone samples collected in the Kemp Caldera. The abundances estimated from the specimens sampled from the collected whale bones ranged from 470 – 6,000 specimens per m^2^, which is within the ranges calculated by Amon et al. [8] for peracarids (lysianassid amphipods and *Jaera tyleri* sp. nov.) on the whale skeleton based on image analysis. Amon et al. [8] reported the peracarid abundances to be higher than the ones of lepetodrillid, osteopeltid and pyropeltid gastropods and siboglinid *Osedax* polychaetes. The abundances of the munnopsid *Ilyarachna profunda* from the southern Californian whale falls are lower and these had also been found in even fewer numbers on the deep-sea sediment and near seeps [6].

The feeding ecology of *Jaera tyleri* sp. nov. is still unknown. The shallow-water congeneric species are herbivores [26], detritivores [23], [25], [27], even cannibals to some extent on weak or dead conspecifics [17], [18] and probably bacterivores [24], [26]. Amon et al. [8] report the abundance of peracarids on the whale bones to be positively correlated with the presence and percentage of bacterial mat cover on the bone and frequently seen on trunks and pulps of *Osedax* sp. nov. This indicated the potential of grazing on the bacteria but does not exclude the digestion of bone itself. Stable isotope or stomach content analyses might be able to clarify this. Within the Janiridae, species of genus *Caecijaera* are small commensals of the wood-borer *Limnoria*; they live in *Limnoria*'s burrows and may feed on the fungi and bacteria living in the burrow walls [57]. Species of another closely related genus, *Iais* are ectocommensal of large sphaeromatids which may not be a specialised wood-borer, but at least they live in burrows of submerged decaying wood [58]. *Jaera hopeana* shares this habit [26]. Wolff [20] reported the presence of janirid isopods in sunken hollow sea grass rhizomes from the bathyal Caribbean trenches.

The observed female-biased sex ratio in *J. tyleri* sp. nov. (1:1.17) is in agreement with analyses on northern hemisphere *Jaera* species [19], [31], although Piertney and Carvalho [59] reported up to twelve times more *J. albifrons* females found at a site in South Wales, United Kingdom. In her 20 months study on *J. nordica* and species of the *J. albifrons* group, Sjöberg [28] discovered a monthly fluctuation in sex ratios from 0.8:1 (males: females) in spring (March) to 2:1 in late autumn (October) after large specimens and ex-ovigerous females had died. Female-biased sex ratios have also been reported from other invertebrate taxa observed at whale falls e.g. polynoid polychaetes [60].

The analyses of the size-frequency distributions of *J. tyleri* sp. nov. suggest multimodal population structure (Fig. 8) with continuous breeding activity throughout the year. The studied northern hemisphere shallow water species of *Jaera* had shown synchronised reproduction with one annual cohort [31], [32]. For Antarctic shelf isopods, Wägele [61], [62] proposed extended reproductive cycles of up to 32 months and seasonally synchronised reproduction.

The fecundity observed in *Jaera tyleri* sp. nov. increases with female size, in line with observations on northern hemisphere *Jaera* species [19], [31], [32]. In the northern species, ovigerous females were found at smaller sizes, carrying more eggs in comparison to the newly discovered species (5–11 eggs observed). In *J. hopeana* 7 –8 eggs and embryos in females of 1.8 mm length were observed [19], while in *J. albifrons* higher fecundity was observed, with 8 – 84 eggs per females [19], [28]. Similar fecundity has been reported for *J. ischiosetosa,* with 5 – 30 eggs per female (2.1–4.8 mm length) [32] and *Jaera nordica, with* 5–28 eggs per female (1.5–4 mm length) [31].

In Isopoda in general the clutch size of females depends on the size of the female. For example, in *Jaera albifrons* a 3 mm large female produces 25 eggs, and a 5 mm large female 64 eggs. An *Idotea* species from the North Sea of 7.5 mm length produces 25 eggs, an 18 mm long female of the same species 324 eggs. In parasitic isopods the numbers of eggs are very high, for example *Cymothoa oestrum* produces around 2500 eggs. However, not all eggs of this ectoporasite develop well and produce offspring, usually about half of the eggs are lost during the brooding process. The breeding time strongly correlates with temperature [63]. A decrease in clutch size with decreasing temperatures, e.g. in cold waters of Antarctica and the deep sea, has been reported [61], but increased fecundity has been observed near hydrothermal vents for invertebrates [64]. In comparison with other Antarctic and deep-sea isopod species, the reported fecundity of *J. tyleri* sp. nov. is similar. In the Antarctic serolid *Ceratoserolis trilobitoides* (Eights 1833) broods of 50 – 170 eggs were observed in females of 42 – 80 mm length, but no correlation between clutch and female size was found [61], [65].

With a single sampling event during the Antarctic summer, nothing can be said about seasonality in the breeding cycle. The presence of juvenile manca stages together with adults and the presence of several cohorts in the size-frequency distributions of immature and mature males and females suggest that the species lives for several breeding cycles. Some northern hemisphere species of *Jaera* showed strong seasonality of their breeding cycles with juveniles being released at certain months while these times vary between species [22], [31], [32]. Others, e.g. species of the *Jaera albifrons* group, breed throughout the year [66]. Sjöberg [28] postulated that *J. albifrons* usually die within a year and undertook one breeding cycle only. Harrison [67] found no evidence for seasonal activity in deep-sea asellote isopods from the north-east Atlantic Rockall Trough, but a strong signal in breeding intensity. More females were breeding through the winter months and more juveniles released during the summer months when the deposition of organic detritus began [67].

## Supporting Information

Figure S1 **The tree from the RaXML secondary structure model 16A analysis of partial 18S rDNA (680 bp).** The tree is rooted on the Stenetriidae. The numbers represent bootstrap support with 100 replicates.
(TIF)
