# Correction: Shallow-Water Northern Hemisphere *Jaera* (Crustacea, Isopoda, Janiridae) Found on Whale Bones in the Southern Ocean Deep Sea: Ecology and Description of *Jaera tyleri* sp. nov

**DOI:** 10.1371/journal.pone.0101092

**Published:** 2014-06-17

**Authors:** The PLOS ONE Staff

Figure 3 is illegible in the XML and PDF of the article. Please see the correct version of Figure 3 below.

**Figure 3:**
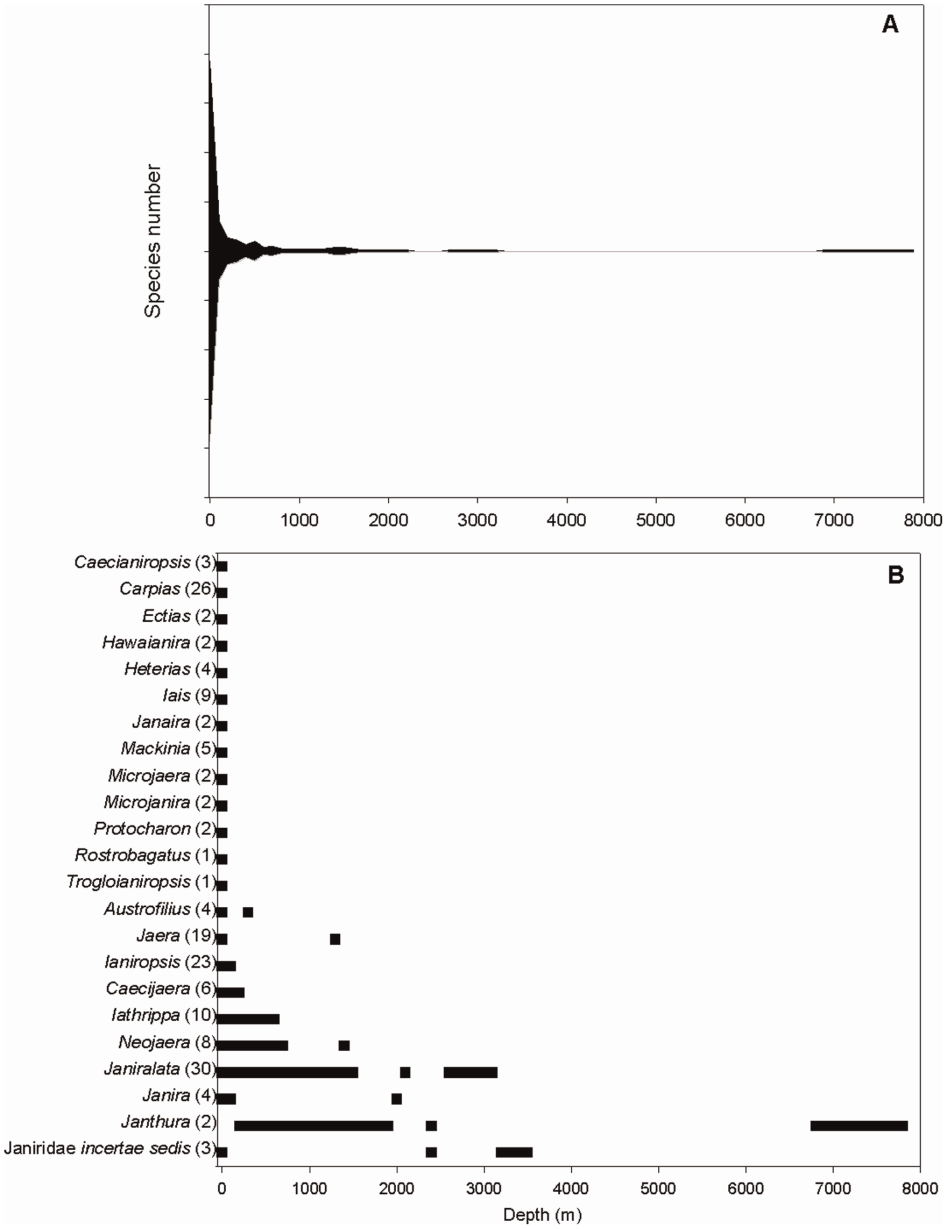
Depth distribution in Janiridae. A) Species numbers per depth. The abscissa divides the total number of species in half, thus the maximum richness is just under 155 species; B) Bathymetric ranges of janirid genera; in bracketsare the numbers of species per family.
