# Supplementary figures and images for: Shallow-Water Northern Hemisphere Jaera (Crustacea, Isopoda, Janiridae) Found on Whale Bones in the Southern Ocean Deep Sea: Ecology and Description of Jaera tyleri sp. nov
Source: PLoS One. 2014 Mar 24;9(3):e93018. doi: 10.1371/journal.pone.0093018 (PMC3963986; doi:10.1371/journal.pone.0093018)

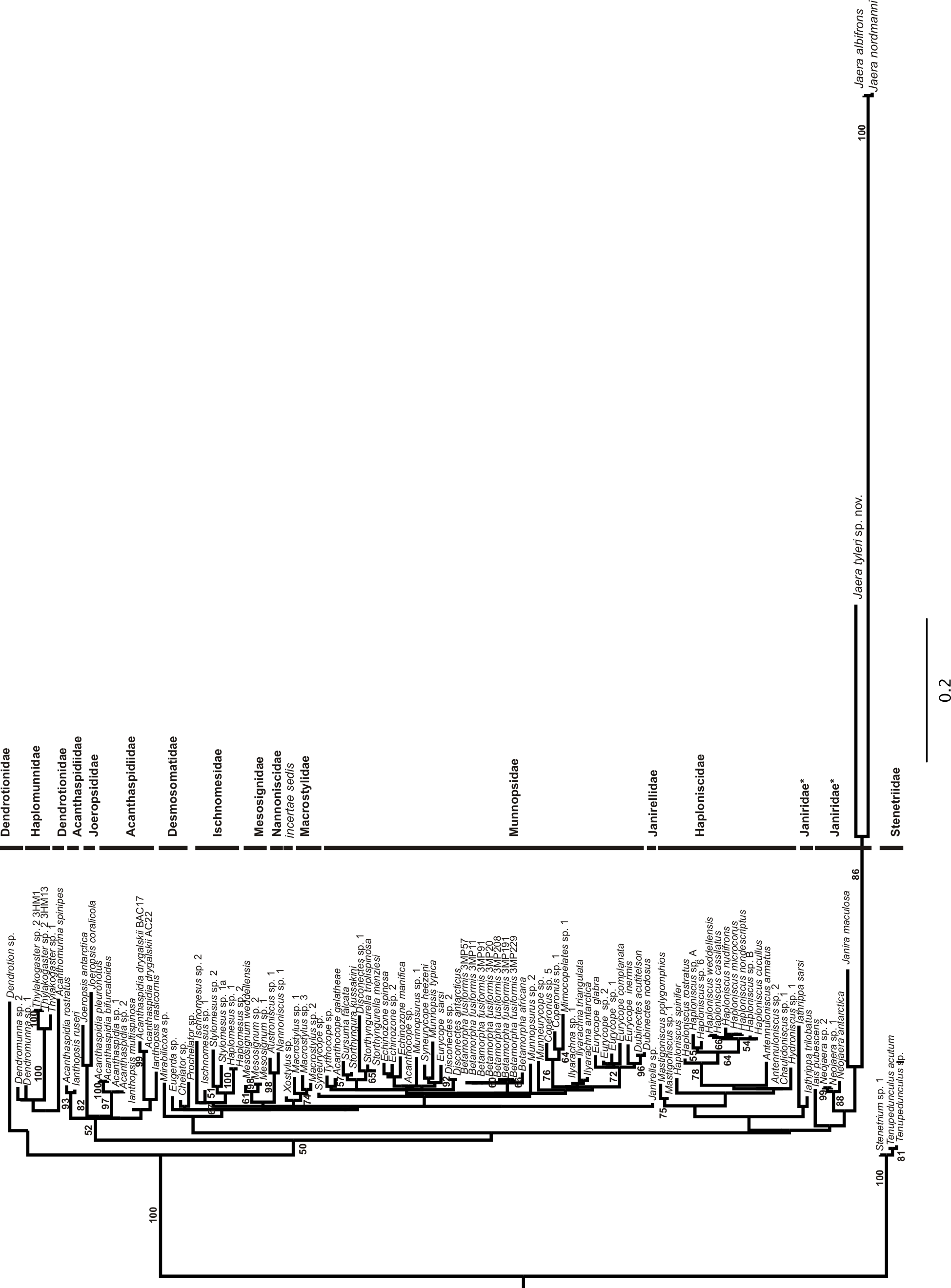

Supplement: Figure S1 — The tree from the RaXML secondary structure model 16A analysis of partial 18S rDNA (680 bp). The tree is rooted on the Stenetriidae. The numbers represent bootstrap support with 100 replicates. (TIF) [file pone.0093018.s001.tif]
